# Diastereoselective Transfer Hydrogenation of Cyclic and Bicyclic Ketones over Selected Metal Oxides as Catalysts

**DOI:** 10.3390/molecules30102153

**Published:** 2025-05-14

**Authors:** Marek Gliński, Dorota Armusiewicz, Karolina Łukasik-Kwaśniewska, Michał Materowski, Adam Rułka, Ewa M. Iwanek (nee Wilczkowska), Monika Kucharska

**Affiliations:** Faculty of Chemistry, Warsaw University of Technology, Noakowskiego 3, 00-664 Warsaw, Poland; pdkalisz@wp.pl (D.A.); karo.lukasik@gmail.com (K.Ł.-K.); michal.materowski@gmail.com (M.M.); rulka.adam@gmail.com (A.R.); ewa.iwanek@pw.edu.pl (E.M.I.); monika.wozniak25@vp.pl (M.K.)

**Keywords:** catalytic transfer hydrogenation, diastereoselectivity, (bi)cyclic ketones, heterogeneous catalysts, oxide catalysts

## Abstract

The diastereoselectivity of the liquid- and vapor-phase Catalytic Transfer Hydrogenation (CTH) of cyclic ketones: x-methylcyclohexanones (x = 2, 3 or 4), 4-*t*-butylcyclohexanone, and bicyclic ketones: 2-norbornanone, camphor, fenchone, and a tricyclic ketone (2-adamantanone) with secondary alkanols (2-propanol, 2-butanol, 2-pentanol, or 2-octanol) as hydrogen donors in the presence of ten metal oxides as the catalysts was studied. Among the oxides, only four, namely, MgO, ZrO_2_·nH_2_O, ZrO_2_, and Al_2_O_3_, exhibited good or high activity. The reaction products are diastereoisomeric alcohols, the relative ratio of which depends on the structure of the ketone, mode of reaction, temperature, and, in the liquid-phase mode, reaction time. The results of vapor-phase CTH revealed that, in this mode of reaction, the diastereoselectivity to the *trans* isomer is lower than in the liquid phase. For the three x-methylcyclohexanones, the most pronounced difference between the experimental values and reference values was noted for x = 3. For bicyclic ketones, the implementation of heterogeneous catalysts allowed us to obtain a substantial excess of the less favorable diastereomer. In the case of 2-norbornanone, the thermodynamic equilibrium mixture contains 21% *endo* and 79% *exo* alcohols, whereas our product mixtures contained up to 79% of the *endo* isomer.

## 1. Introduction

Chemical processes involving gaseous hydrogen constitute the largest group of chemical reactions both in the laboratory and, more importantly, in the chemical industry. Examples of technologies involving hydrogen include the following: ammonia synthesis, the hydrogenation of carbon monoxide to methanol, synthetic gasoline, aliphatic alcohols or aromatic hydrocarbons (depending on the reaction conditions and the type of catalyst), as well as hydrocracking and hydrodesulfurization (HDS). Most of these processes are carried out worldwide on a scale of tens or even hundreds of millions of tons per year. Apart from numerous advantages, the hydrogenation of chemical compounds with gaseous hydrogen has two pronounced disadvantages. First of all, working with gaseous hydrogen is usually performed under substantial pressure and hence requires the use of pressure equipment. Moreover, due to the flammability of hydrogen, special caution must be taken in installations that utilize this gas. Catalytic Transfer Hydrogenation (CTH) is an alternative method of the hydrogenation of organic compounds and is free from these disadvantages. In this method, compounds called hydrogen donors are used as the source of hydrogen, which, in the presence of a catalyst, are able to transfer a molecule or molecules of hydrogen to the hydrogenated compound, i.e., the hydrogen acceptor. The process does not require the use of elevated pressure and already occurs at moderate temperatures. Various functional groups can be hydrogenated via this method. In recent years, CTH has been a frequently explored research topic [1]. As per data from a 2023 review article, it is estimated that over 285,000 articles on this topic have been published between the years 2000 and 2020 [1], though they are focused mainly on the implementation of homogeneous catalysis and the use of organometallic complexes of transition metals such as Ru [2], Rh [3], Ir, Pd, Mn [4], Co, and Fe [5]. Many papers/reviews on the topic of Homogeneous Transfer Hydrogenation have been recently published [6,7,8,9,10], which show the wide scope of the research in homogeneous transfer hydrogenation in terms of the hydrogen donors: methanol, ethanol, glycerol, carbohydrates, etc.; hydrogen acceptors: α,β-unsaturated and other ketones, aldehydes, alkenes, imines, carboxylic acids, etc.; as well as different catalyst synthesis routes and mechanistic aspects of the reaction. Much less attention has been paid to the studies of heterogeneous CTH with the participation of metal oxides as catalysts, despite the fact that they are much cheaper than noble metals. In the past, the CTH reaction with the participation of heterogeneous catalysts in the form of metallic blacks, metals deposited on a support, metal oxides, and also zeolites was the subject of several review works [11,12,13]. The carbonyl group of aldehydes and ketones is particularly easily reduced by alcohols as hydrogen donors in the CTH reaction using various metal oxide catalysts, among which the most active is magnesium oxide.

For the last twenty years, our team has performed systematic research on the CTH of various carbonyl compounds in the presence of magnesium oxide as the principal catalyst. The following carbonyl compounds have been investigated in our studies:

- Aliphatic ketones with increasing steric hindrance in close proximity to the carbonyl group caused by the introduction of an increasing number of methyl substituents at both α-carbons [14];
- Straight-chained aliphatic ketones with 11 carbon atoms in the chain, in which the carbonyl group was present in different positions along the chain (positions 2–6) [14];
- 1-phenyl-1-alkanones substituted with alkyl groups in the ring and in the side chain [15];
- Diaryl ketones with different substituents present in the aromatic rings [16];
- α,β-unsaturated aldehydes and ketones [17,18];
- 4-*t*-butylcyclohexanone [19];
- 1- and 2-acetylnaphthalenes, and diacylbenzenes [20].

In short, these studies delved into the reactivity of compounds in the CTH reaction depending on their structure, the chemoselectivity of the reduction of the carbonyl group of compounds containing two reducible groups, and also diastereoselectivity in the case of the alkylcyclohexanone derivative. The last of these prompted us to investigate the topic of diastereoselectivity of CTH further.

The problem of diastereoselectivity in the reduction of the carbonyl group of cyclic ketones has been the subject of several studies in the past. In the initial period of research in the 1950s, the focus was on collecting data on the influence of the structure of ketones, the type and structure of reducing agents, and the type of solvents on the course of the reaction. The measurements were carried out in the liquid-phase mode using a ketone and various reducing agents in stoichiometric amounts dissolved in a selected solvent. Metal hydrides were typically used as reducing agents: LiAlH_4_ [21,22], NaBH_4_ [23,24], LiAlH(OCH_3_)_3_ [25], KHBPh_3_ [26], as well as aluminum and lanthanide alkoxides, e.g., Gd(O-*i*-C_3_H_7_)_3_ [27] and solutions of alkali metals (Li, Na, K, and Rb) in liquid ammonia [28]. Later research on diastereoselectivity in the transfer hydrogenation of ketones focused on the application of heterogeneous catalysis. In the work of Ramana and Pillai, the transfer hydrogenation of (−)-menthone to a mixture of menthol and neomenthol, by different alcohols in the presence of alumina with 2 wt.% of sodium ions, was performed at 573 K in the vapor phase. It was shown that the neomenthol content in the post-reaction mixture was substantially influenced by the type of hydrogen donor used [29]. Shibagaki et al. used hydrous zirconium oxide as the catalyst in vapor-phase CTH of 2-methyl-, 4-methylcyclohexanone, and camphor with 2-propanol [30]. For the first two ketones, they reported a quantitative conversion and a *trans*/*cis* molar ratio of the diastereomeric alcohols of 44:56 and 73:27 for 2-methyl- and 4-methylcyclohexanone, respectively, at 373 K. In the case of camphor reduced at 423 K, a conversion of 64% was reported, and the post-reaction mixture contained 59% of *exo* and 41% of *endo* isomer. An increase in reaction temperature (473 K) led to an increase in the conversion (88%). However, under these conditions, camphene was the main product (56%), which was formed by the dehydration of the obtained alcohols. Zeolite BEA (Si/Al = 12) has been studied as a catalyst in the CTH of 4-*t*-butylcyclohexanone with 2-propanol [31]. A high diastereoselectivity (>95%) to the thermodynamically less stable *cis*-isomer was obtained. This is explained by transition-state selectivity imposed by the zeolite structure. Corma et al. used Al-free Sn-Beta zeolite (2 wt.% SnO_2_) in the CTH of 4-*t*-butylcyclohexanone with 2-propanol or 2-butanol as hydrogen donors. After 6 h of reaction, a conversion of 97% was noted for both donors. What is important is that a very high diastereoselectivity to the *cis* isomer (96–99%) was noted [32]. In another work, the authors used the same catalyst in the CTH of regioisomeric x-methylcyclohexanones, where x = 2, 3, or 4. In total, 100% of *cis* alcohol isomer was reported in the reduction of 4-methyl- and 3-methylcyclohexanones, whereas the reduction of 2-methylcyclohexanone led to a mixture of *cis* and *trans* alcohols with a molar ratio of 50.0:45.4 [33]. Magnesium oxide was subjected to preliminary activity measurements in the CTH of 4-*t*-butycyclohexanone with various secondary alcohols as hydrogen donors in the vapor and liquid phase [19]. In the vapor-phase reaction in the temperature range of 473–623 K, six secondary aliphatic alcohols were used as donors, namely, the following: 2-propanol, 2-butanol, 2-pentanol, 3-pentanol, 4-heptanol, and 5-nonanol. At 473 K, a lower yield of the alcohol mixture was found for the last two donors, 52 and 42%, respectively, compared to the 93% yield recorded for 2-propanol at the same temperature. The diastereoselectivity of the CTH reaction (70% *trans*, 2-propanol) decreased only very slightly with increasing molecular weight of the hydrogen donor (66% *trans*, 5-nonanol). A very significant increase in the diastereoselectivity of the reaction was observed after changing the CTH mode from vapor- to liquid-phase mode. In the case of 2-propanol as the hydrogen donor (D/A = 6), a 95% yield of the alcohol mixture and 97% diastereoselectivity towards the *trans* isomer were observed after 180 min of reaction. Changing the donor to one with a higher boiling point did not increase the yield of alcohols in the reaction, despite the higher reaction temperature, but reduced the diastereoselectivity to 91%, as in the case of 2-octanol as the hydrogen donor. This behavior can be explained by the effect of the temperature on the thermodynamic composition of the mixture of obtained alcohols. What is most intriguing is that, in the CTH reaction in the liquid phase, the composition of the mixture of 4-*t*-butylcyclohexanols, despite their very long contact with the hydrogen donor and the catalyst surface, is far from the alcohol composition obtained in the vapor-phase reaction at only slightly different reaction temperatures, i.e., vapor phase (473 K) and liquid phase (452 K, 2-octanol), with very short contact in the former case.

The aforementioned studies have not yet allowed a meaningful comparison of the influence of the CTH reaction conditions on diastereoselectivity due to the lack of systematic tests with each variable tested separately, namely, the following: change in oxide catalyst, change in mode of reaction, change in acceptor, change in donor, and reaction temperature. Table 1 lists the different conditions of studies thus far performed in which the hydrogen donor was an alcohol or a metal hydride, and the hydrogen acceptor was a cyclic or bicyclic ketone. It can be seen that most of the studies have been carried out in the liquid phase mode of reaction, and there are very few studies performed in the vapor phase, which is why our study includes both modes of reaction for each of the studied hydrogen acceptors. 

Moreover, it should be noted that the donors studied in the reactions are typically few, and, hence, a broader range of chain lengths could provide meaningful insight into the studies, which is the reason for carrying out our present study with four different donors, namely, the following: 2-propanol, 2-butanol, 2-pentanol, and 2- octanol. When catalysts were used in these studies (Table 1), it was usually one or two samples per paper, and either amorphous (ZrO_2_·nH_2_O) or crystalline (Al_2_O_3_ + 2 wt% Na) materials were used. This is why our paper presents the results of a systematic study of the activity of several crystalline oxides that vary in the surface properties and two amorphous oxides, i.e., SiO_2_ and ZrO_2_·nH_2_O, which enables us to gain insight into how the surface properties influence the diastereoselectivity of these reactions. A crystalline ZrO_2_ sample was also used as a reference for the ZrO_2_·nH_2_O catalyst, which has been reported to exhibit high activity by other research groups.

The preliminary studies conducted by us several years ago with MgO as a catalyst proved the suitability of this method for reducing 4-*t*-butylcyclohexanone to *trans* 4-*t*-butylcyclohexanol with a yield as high as 97% after 180 min of reaction and with a diastereoselectivity of 97–98% [19]. A new, valuable aspect not present in the reduction in ketones with a different structure is the possibility of determining the side from which the attack of the reducing agent on the carbonyl group occurs. It is well known that this attack from the axial position results in the appearance of a hydroxyl group in the equatorial position. Thus, the use of 10 different metal oxides as catalysts allowed us to compare their activity and, above all, the diastereoselectivity of the reduction in the ketones studied in order to attempt to link the properties of the catalysts used with their activities and diastereoselectivities observed in the CTH reaction.

## 2. Results and Discussion

### 2.1. Characterization of Catalysts

#### 2.1.1. Scanning Electron Microscopy Coupled with Energy-Dispersive X-Ray Spectroscopy (SEM-EDX)

**Figure 1:**
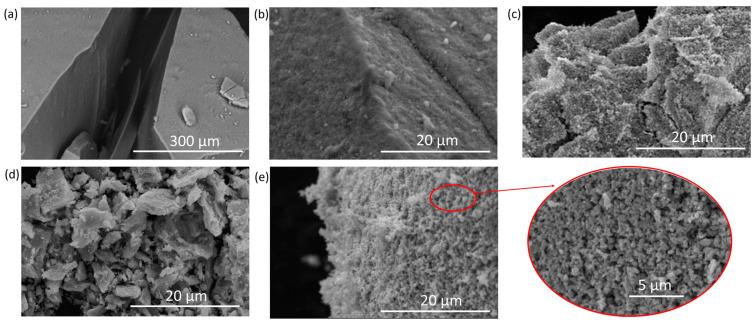
SEM images of (**a**) SiO_2_, (**b**) TiO_2_, (**c**) Cr_2_O_3_, (**d**) CeO_2_, and (**e**) ZnO at different magnifications.

The topography of the grains of most of the oxides, namely, the following: SiO_2_, Al_2_O_3_, TiO_2_, ZrO_2_, and ZrO_2_·nH_2_O is very similar: at a low magnification, the particles are shaped like chipped blocks (Figure 1a), whereas, at a higher magnification (Figure 1b), they are dense slabs with small particles on top, which form upon a fracture of the oxide chunks while obtaining the proper fraction size for the catalytic reactions. The three oxides that exhibit a different topography are as follows: Cr_2_O_3_ (Figure 1c) and CeO_2_ (Figure 1d), both of which appear to be formed of smaller pieces than those of the aforementioned oxides, and ZnO (Figure 1e), which is composed of much finer particles, as seen in the insert.

**Figure 2:**
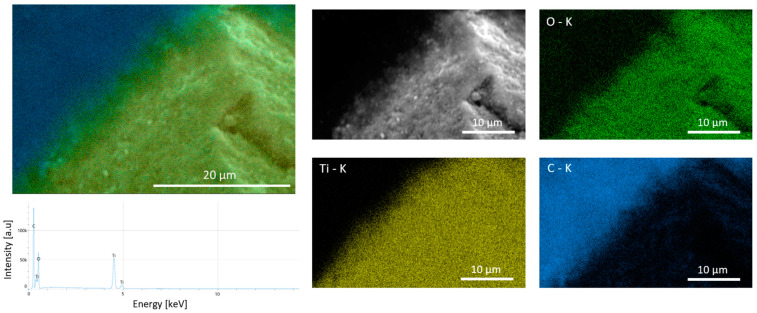
ChemiSEM^TM^ image, SEM image, EDX elemental maps obtained for TiO_2_, and the EDX spectrum.

The EDX analysis of the oxides (Figure 2) shows that, in the spectrum of a given oxide, e.g., TiO_2_, there are three main components: carbon, which is present in abundance in the part of the image outside of the grain because they were taped onto the stub using carbon tape (see Section 3), and the pure oxide, which consists of the two elements, i.e., oxygen and the metal cation (in this case titanium). In the elemental map, it can be seen that the titanium is not present in the right top area on the carbon tape, whereas some oxygen is also present on the carbon tape, which is typical. No other elements are present in the EDX spectra of the samples (Figure 2).

**Figure 3:**
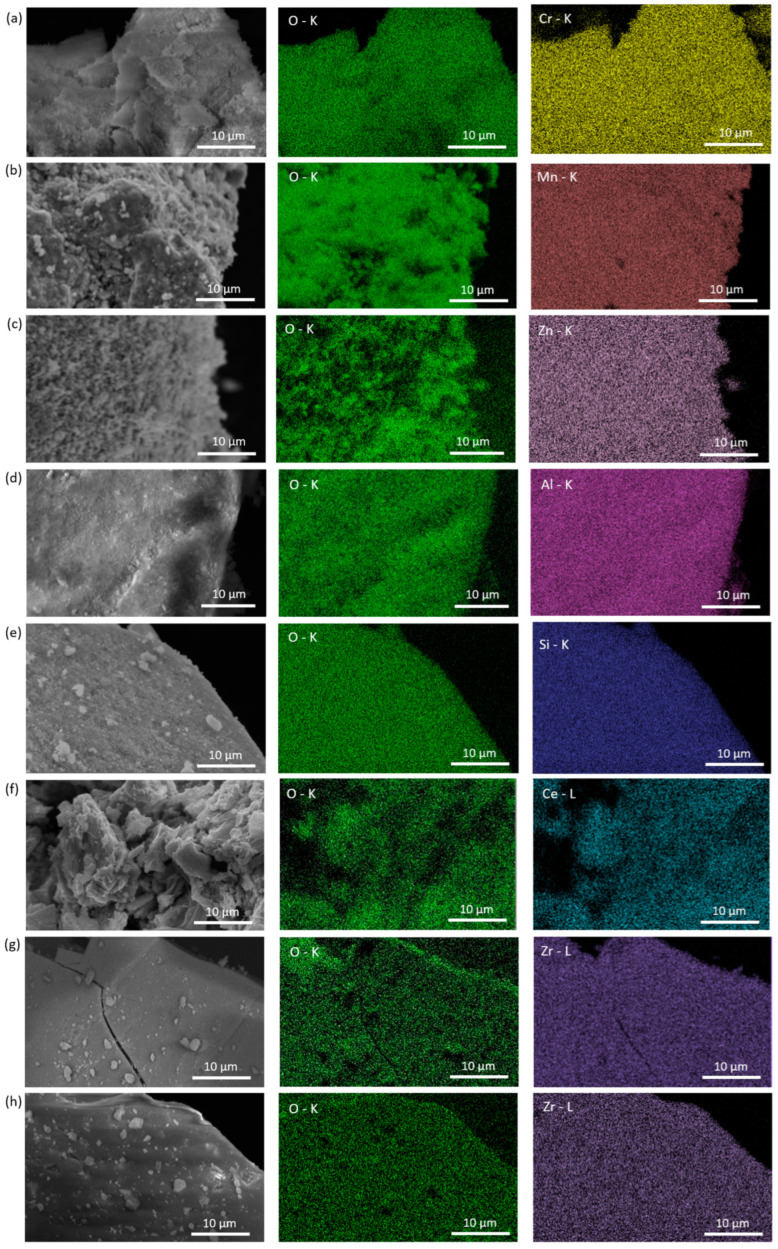
EDX results: Elemental maps of the oxide catalysts: (**a**) Cr_2_O_3_, (**b**) MnO_2_, (**c**) ZnO_2_, (**d**) Al_2_O_3_, (**e**) SiO_2_, (**f**) CeO_2_, (**g**) ZrO_2_, and (**h**) ZrO_2_·nH_2_O.

The elemental maps and SEM images of all of the other studied oxides are compiled in Figure 3. As in the case of TiO_2_, it can be noticed that the elemental map of oxygen in all studied oxides shows much more detail than that of the counter ion. This is typical for heavier metals. The SEM image and elemental maps show very little difference between ZrO_2_ (Figure 3g) and ZrO_2_·nH_2_O (Figure 3h), though the latter is amorphous as shown in the XRD pattern and in the DTA-TGA measurements (Supplementary Materials, Figure S1a,b), which clearly indicate the loss of constituent water (approx. 8 wt.%), followed by an exothermic peak that is attributed to crystallization as reported by Picquart et al. [34]. Based on the mass loss obtained during TGA measurements, it was found that the composition of the hydrate of this oxide corresponds to the formula ZrO_2_·0.60 H_2_O. This change, however, occurs at K and hence is not expected under reaction conditions. It is noteworthy that the other amorphous oxide (SiO_2_) does not show signs of crystallization in the studied range of temperatures (Figure S1c).

The mass loss curve of MnO_2_ (Figure S1d) shows a distinct drop at temperatures above 800 K along with an endothermic effect, which is attributed to the thermal decomposition of this oxide to Mn_2_O_3_. The thermal analysis of MgO (Figure S1e) shows the desorption of residual physiosorbed water as well as chemically bound water (in the form of hydroxide), which decomposes in the range 500–600 K and a steady mass at higher temperatures. In the case of Cr_2_O_3_ (Figure S1f), it can be seen that, at the end of the measurement, there is a beginning of a mass loss, which accounts for less than 1 wt.%. The last four oxides do not reveal any changes in the entire range of studied temperatures; the only differences in the curves result from differences in thermal capacity and oxide density (Figure S1g–j).

#### 2.1.2. Powder X-Ray Diffraction (PXRD) and Nitrogen Physisorption

**Figure 4:**
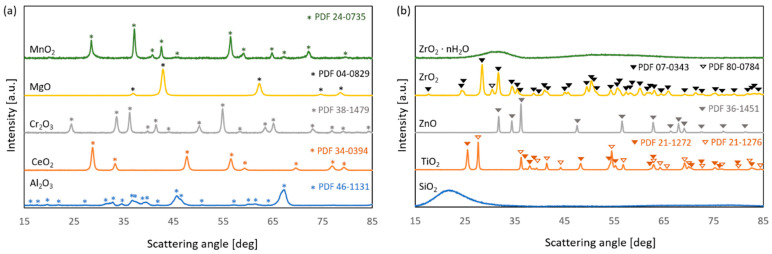
Diffraction patterns of the metal oxide catalysts: (**a**) Al_2_O_3_, CeO_2_, Cr_2_O_3_, MgO, and MnO_2_, (**b**) SiO_2_, TiO_2_, ZnO, ZrO_2_, and ZrO_2_·nH_2_O.

Ten metal oxides (Al_2_O_3_, CeO_2_, Cr_2_O_3_, MgO, MnO_2_, SiO_2_, TiO_2_, ZnO, ZrO_2_, and ZrO_2_·nH_2_O) were examined using the PXRD method (ex situ measurements). The main phases identified in the studied catalysts are indicated in Figure 4a,b. It can be seen that they are pure, as no other reflexes from any other phase have been detected. Among the tested metal oxides, two of them, SiO_2_ and ZrO_2_·nH_2_O, were amorphous. Titanium dioxide was a mixture of two polymorphs: rutile and anatase, whose relative ratio is 1.4:1. Crystalline zirconium dioxide was also a mixture of two polymorphs, with the monoclinic phase as the main component and a small fraction of the tetragonal phase. Based on Scherrer’s formula (for k = 0.89), the sizes of crystallites in the case of crystalline oxides were calculated. The results are summarized in Table 2 along with the specific surface area values of the oxides. All crystalline phases consisted of crystallites with an average size between 12 and 33 nm.

A large variation in the values of the specific surface area of the tested metal oxides was observed. The highest surface area was noted for the two amorphous oxides, namely, SiO_2_ and ZrO_2_·nH_2_O. The values were 253 and 235 m^2^·g^−^^1^, respectively. It is noteworthy that the crystalline ZrO_2_ has a surface area of only 32 m^2^·g^−^^1^. The two crystalline catalysts with the highest surface area are MgO (100 m^2^·g^−^^1^) and Al_2_O_3_ (103 m^2^·g^−^^1^). The fact that the surface of these is practically the same in terms of area but is known to significantly differ in the basic/acidic properties implies that a meaningful assessment of how the latter impacts the activity will be possible. Both crystalline ZrO_2_ and TiO_2_ are present in two polymorphic forms and have very similar surface areas. Three of the samples exhibit surface areas under 10 m^2^·g^−^^1^. They are CeO_2_, ZnO, and MnO_2_ (Table 2).

#### 2.1.3. Acid/Base Properties

The acid/base properties of the studied oxides were conducted using two methods. The first of these is the use of Hammett indicators, which allow the determination of the range of strengths of the acidic and basic sites (Table 3). All of the indicators used are listed in Section 3. The indicators, which changed color in the presence of the appropriate sites of a studied oxide, are shown with a red plus, whereas the lack of reaction with the indicator was denoted by a minus. It can be seen that the alumina used in this study possesses mostly acidic sites, whereas its basicity is very limited. In contrast, the obtained magnesia exhibits exclusively basic sites. In fact, it has the widest range of basic sites out of all the studied oxides. The fact that it reacted with the 26.5 indicator qualifies it, in accordance with Tanabe’s definition, as a superbase.

A slightly narrower range of the strength of basic sites was noted for ZrO_2_·nH_2_O (Table 3). This is interesting considering the fact that the anhydrous zirconia sample has an H_−_ range of only 7.2 ≤ H_−_ < 18.4. It is noteworthy that both zirconia catalysts additionally exhibit acidic sites, although, again, the hydrous oxide possesses a wider range than the anhydrous one. Both alumina and ZrO_2_·nH_2_O have a range of acidic sites whose strength can be described by the following: −5.6 < H_0_ ≤ 4.8. ZrO_2_ and CeO_2_ have the same range of strength of both types of sites (Table 3). The ranges exhibited by titania and ZnO are narrower, with the former being more acidic and the latter more basic in nature. SiO_2_ is the most neutral of the studied oxides, which is indicated by the presence of only the weakest acidic and basic sites.

The acidic and basic nature of the surfaces of the studied oxides was further probed by titration with n-butylamine and benzoic acid, respectively. In Table 4, the order of the concentration of acidic sites (expressed in µmol·m^−2^) on the surface of oxides is as follows: MnO_2_ (21.4) > CeO_2_ (17.3) > ZnO (10.2) > Cr_2_O_3_ (8.6) > TiO_2_ (4.2) > ZrO_2_ (3.2) > Al_2_O_3_ (2.3) > ZrO_2_·nH_2_O (1.7) > SiO_2_ (1.3) > MgO (0.8). The order of diminishing concentration of basic sites is as follows: CeO_2_ (26.7) > MgO (11.7) > ZnO (8.1) > Al_2_O_3_ (5.3) > ZrO_2_ (3.7) > TiO_2_ (3.3) > ZrO_2_·nH_2_O (3.1) > Cr_2_O_3_ (2.4) > MnO_2_ (0.2) > SiO_2_ (~0.0). It is noteworthy that CeO_2_ shows a very high concentration of both acidic and basic sites and that the concentration of basic sites on the surface of SiO_2_ is close to zero. The order is different when taking into account the mass rather than the surface area. Per gram of catalyst, the amorphous ZrO_2_·nH_2_O exhibits the highest concentration of acidic sites, and MgO possesses much more basic sites than any other oxide. The reference to the surface area is more meaningful in the case of catalysts because the surface is where the reaction occurs.

### 2.2. Activity Measurements

In our studies on the diastereoselectivity of Catalytic Transfer Hydrogenation, the following eight ketones were used as hydrogen acceptors: (1) four monocyclic ketones: 2-methyl-, 3-methyl-, and 4-methylcyclohexanone as well as 4-*t*-butylcyclohexanone; (2) three bicyclic ketones: 2-norbornanone (bicyclo [2.2.1]heptan-2-one), camphor (1,7,7-trimethylbiclo[2.2.1]heptan-2-one), and fenchone (1,3,3-trimethylbicyclo[2.2.1]heptan-2-one); and (3) one tricyclic ketone: 2-adamantanone (tricyclo[3.3.1.1^3,7^]decan-2-one). Four secondary alcohols, namely, 2-propanol, 2-butanol, 2-pentanol, and 2-octanol, were used as hydrogen donors. Ten metal oxides (SiO_2_, ZrO_2_·nH_2_O, Al_2_O_3_, MgO, TiO_2_, ZrO_2_, Cr_2_O_3_, CeO_2_, ZnO, and MnO_2_) were applied as catalysts both in liquid- and vapor-phase modes of reaction. The structures of all of the product molecules can be seen in Scheme S1 in the Supplementary Materials.

The presence of alkyl substituents in cyclohexanone molecules implies that, after reduction of their carbonyl groups, the substituted cyclohexanols formed in this way are no longer conformations but become specific configurations. This is due to significant differences in the stability of individual potential conformations resulting from different interactions of alkyl substituents in equatorial or axial positions of the cyclohexane ring. This view, presented by Winstein and Holness, was of great importance for understanding the course of the reduction in cyclic ketones, according to which, the introduction of a bulky alkyl group, such as *t*-butyl, into the cyclohexane molecule prevents conformational transformations of the ring and leads to the formation of two diastereomeric 4-*t*-butylcyclohexanols as a result of the reduction of 4-*t*-butylcyclohexanone [35].

In contrast to thermodynamic calculations, the Catalytic Transfer Hydrogenation of ketones is kinetically controlled. Therefore, the thermodynamic equilibrium composition can only serve as a reference value. In this study, the discrepancies between the obtained values and the reference values were used as a valuable tool to compare the diastereoselectivity of the reactions on different catalysts. In the case of the reduction of 4-*t*-butylcyclohexanone, the equilibrium composition of the 4-*t*-butylcyclohexanol mixture was discussed by Winstein and Holness [35] and was found to contain 78% *trans* and 22% *cis* diastereomers. The methyl substituent in 4-methylcyclohexanone, which is smaller than the *t*-butyl group, can occupy either the equatorial or axial position. In an equilibrium post-reduction mixture, the fraction of *trans* alcohol (70 ± 1%) is slightly lower than that determined for 4-*t*-butylcyclohexanols [36]. The composition of alcohols formed by the reduction in cyclic ketones without steric hindrance caused by the presence of bulky substituents is primarily determined by the relative stability of isomeric alcohols, i.e., the thermodynamic factor. In contrast, in the case of bicyclic ketones containing a methylene bridge that stiffens the molecule, such as in 2-norbornanone, camphor, fenchone, thujone, and others, the composition of the mixture of stereoisomeric alcohols formed in their reduction is primarily determined by the ease of access of the reducing agent to the carbonyl group, i.e., the kinetic factor. In the case of 2-adamantanone, containing three fused cyclohexane rings, the transfer hydrogenation to its carbonyl group results in the formation of 2-adamantanol, which does not cause any stereochemical effects due to the high symmetry of the parent hydrocarbon molecule.

#### 2.2.1. Liquid- and Vapor-Phase Transfer Hydrogenation of 4-*t*-Butylcyclohexanone

**Scheme 1:**
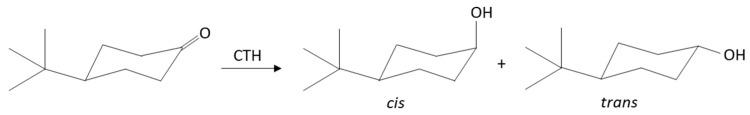
4-*t*-butylcyclohexanone and two diastereoisomeric 4-*t*-butylcyclohexanols.

The transfer hydrogenation reaction from 2-propanol to 4-*t*-butylcyclohexanone is shown in Scheme 1. In this reaction, two diastereoisomeric 4-*t*-butylcyclohexanols are formed, with the *trans* isomer being more stable. Under conditions of thermodynamic equilibrium, the ratio of *trans*/*cis* isomers of these alcohols is 78:22 [36]. Ten metal oxides were used as the catalysts in the liquid-phase transfer hydrogenation of 4-*t*-butylcyclohexanone with 2-octanol. This alcohol was chosen for the preliminary experiments because it has the highest boiling point (452 K) among the alcohols used in the work. It was expected that the highest conversions of 4-*t*-butylcyclohexanone would be achieved in its presence. The results are summarized in Table 5.

**Scheme 2:**
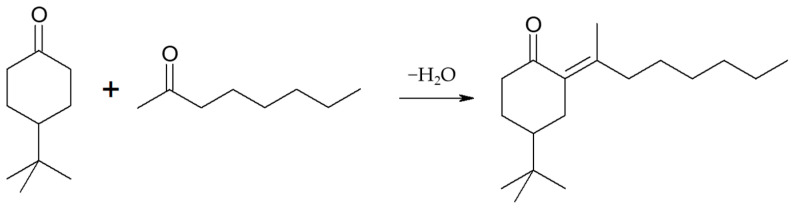
Reaction between 4-*t*-butylcyclohexanone and 2-octanone formed in the main reaction.

The catalysts in Table 5 are arranged in order of increasing activity expressed in conversion values. In the group of metal oxides with low (less than 30%) activity in the reaction, the activity determined after 6 h ranges from 2 to 23%. The order of increasing activity in this group is the percentage value of conversion given in brackets: SiO_2_ (2) < CeO_2_ (4) < ZnO (9) < MnO_2_ (10) < TiO_2_ (12) < Cr_2_O_3_ (23). Among the metal oxides of this group, only SiO_2_ is a representative of oxides of the main group elements, and the rest are oxides of transition metals. Moreover, it is noteworthy that this oxide possesses the lowest reaction activity despite having the highest specific surface area (Table 2). For this group of catalysts, the selectivity towards the formation of a mixture of alcohols (4-*t*-butylcyclohexanols) after 3 h of reaction was 100% and decreased to approximately 90% after 6 h. The decrease was caused by the side reaction between 4-*t*-butylcyclohexanone and 2-octanone formed in the main reaction. The side reaction is depicted by Scheme 2.

The diastereoselectivity of the reaction to the thermodynamically more stable *trans* diastereoisomer can be expressed as [100 *trans*/(*trans* + *cis*)%]. Under conditions of thermodynamic equilibrium, the mixture of 4-*t*-butylcyclohexanols contains 78 ± 1% of the *trans* isomer. In the case of ZnO and MnO_2_, it was found that, in their presence, the fraction of the *cis* isomer in the 4-*t*-butylcyclohexanols mixture is much higher than the reference value. The value obtained for ZnO (58%) is noteworthy, as it indicates an almost equimolar composition of alcohols.

In the group of highly active catalysts (ZrO_2_, ZrO_2_ ·nH_2_O, Al_2_O_3_, and MgO), conversions above 72% were recorded after just 1 h of reaction. Very high conversion values of 4-*t*-butylcyclohexanone are accompanied by high or very high yields of the 4-*t*-butylcyclohexanol mixture. The selectivity of the reaction to 4-*t*-butylcyclohexanols depends on the choice of catalyst. For Al_2_O_3_, it was observed that a relatively low yield of 4-*t*-butylcyclohexanols (66%) was obtained despite the high conversion (96%). This is mainly due to the dehydration of 4-*t*-butylcyclohexanols, which occurs quickly at the reaction temperature (452 K) in the presence of Brønsted acid sites present on the Al_2_O_3_ surface. MgO exhibited the highest activity in this group of catalysts; in its presence, 98% conversion and 71% diastereoselectivity to the *trans* isomer were recorded after 3 h of reaction. An 88% selectivity to 4-*t*-butylcyclohexanols indicates the occurrence of a side reaction. Due to the presence of strong basic sites on the MgO surface, this side reaction is the aldol condensation of 4-*t*-butylcyclohexanone with 2-octanone formed in the main reaction. The primary condensation product (aldol) undergoes a subsequent dehydration reaction to form an α,β-unsaturated carbonyl compound, as shown in Scheme 2.

The catalytic activity of all four metal oxides from the second group of catalysts was tested in the transfer hydrogenation reaction using 2-propanol as a hydrogen donor. The results of these studies are summarized in Table 6. It was found that changing the hydrogen donor from 2-octanol to 2-propanol and thus lowering the reaction temperature from 452 to 355 K resulted in differences in the conversion of 4-*t*-butylcyclohexanone in the group of the most active catalysts. ZrO_2_ turned out to be the least active catalyst after 3 h of reaction. In its presence, only 47% conversion was recorded. In the case of the remaining oxides, the decrease in conversion, associated with the lower reaction temperature, was smaller. It is noteworthy that the selectivity to 4-*t*-butylcyclohexanols in the case of Al_2_O_3_ was 99–100%. At this temperature (355 K), the subsequent dehydration reaction of the primary products of the transfer hydrogenation reaction practically does not occur. MgO remained the most active oxide in the reaction, with a conversion of 86%, and with high (>95%) selectivity to alcohols. It was found that, at 355 K, the diastereoselectivities of the reaction to the *trans* isomer for all catalysts were higher than those observed at the higher temperature (452 K). This is due to the equalizing effect of temperature increase on the stability of alcohol isomers. The highest value of diastereoselectivity to the *trans* isomer (97%) was observed in the case of MgO (Table 6).

**Figure 5:**
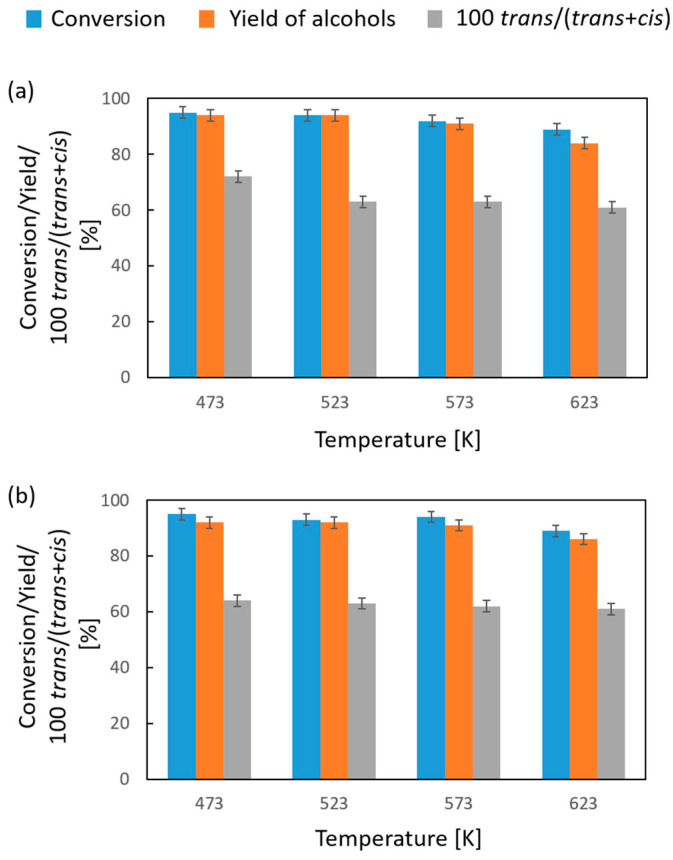
Vapor-phase transfer hydrogenation of 4-*t*-butylcyclohexanone with 2-propanol in the presence of (**a**) fresh MgO and (**b**) spent MgO catalyst.

A study on the transfer hydrogenation reaction in the vapor phase from 2-propanol to 4-*t*-butylcyclohexanone was also carried out in the presence of MgO, the most active catalyst as per the previous part of our work. The results are summarized in Figure 5. Already at a temperature of 473 K, a 95% conversion of 4-*t*-butylcyclohexanone was recorded, which decreased slightly with an increase in the reaction temperature to 89% at 623 K. A similar profile of changes in ketone conversion was observed in the case of a test with the spent catalyst, which clearly excludes its deactivation as the reason for the reduction in conversion with the reaction temperature. In the test with a fresh catalyst, similarly to the conversion, the selectivity to alcohols and diastereoselectivity decreased with temperature. As for selectivity, its decrease from 97 to 94% was the result of aldol condensation between 4-*t*-butylcyclohexanone and acetone formed in the CTH reaction, whereas the decrease in diastereoselectivity of the reaction resulted from the equalizing effect of temperature on the molar ratio of diastereoisomeric 4-*t*-butylcyclohexanols. What is noteworthy is the significantly lower value of diastereoselectivity of the reaction to the *trans* diastereoisomer in the vapor phase, which is 61–63% compared to the value obtained in the liquid phase with the same hydrogen donor (97%). This value is a result of two factors, namely, the following: the high reaction temperature in the vapor phase and the mechanism of the reaction. It is commonly accepted that Catalytic Transfer Hydrogenation over MgO [37] and other catalysts [27,31,32,33] occurs via the Meerwein–Ponndorf–Verley mechanism, whose key step is the transfer of a hydride anion from the carbinol atom of the donor to the carbonyl group of the acceptor. Therefore, the stereochemistry of the attack of the hydride anion on the carbonyl carbon in the ketone molecule also impacts the *cis/trans* ratio. There is no doubt that the adsorption of 4-*t*-butylcyclohexanone on the MgO surface involves the interaction of carbonyl oxygen with the Lewis acid site, which is the surface magnesium cation. Under these conditions, the *t*-butyl group in the equatorial position is protruding from the surface. Hence, the attack of the hydride anion from an axial position is very strongly favored. In the CTH of the 4-*t*-butylcyclohexanone reaction in the vapor phase, there are two explanations for the significantly lower diastereoselectivity of the reaction to the *trans* stereoisomer. In the first one, it is assumed that, after adsorption of the donor on the catalyst surface, the ketone attacks from the vapor- phase and this attack take place in a statistical manner, because the bulk substituent in the form of the *t*-butyl group located in position 4 does not create a significant steric hindrance for the attack of the carbonyl carbon atom located in position 1. In the second explanation, it should be assumed that the CTH reaction is fast and reversible under the reaction conditions, and this causes the composition of 4-*t*-butylcyclohexanols to be equilibrated.

#### 2.2.2. Liquid- and Vapor-Phase Transfer Hydrogenation of x-Methylcyclohexanones (x = 2, 3 or 4)

In the second part of our research, we focused on variously substituted mono-methylcyclohexanones. Depending on the relative position of the methyl group and the carbonyl group in the cyclohexane ring, the diastereoisomeric alcohols formed as a result of CTH differ in their stability. In the case of the reduction of 4-methylcyclohexanone, the *trans* isomer of 4-methylcyclohexanol is the thermodynamically more stable one. Under equilibrium conditions, the composition of the mixture of 4-methylcyclohexanols is given as [100 *trans*/(*trans* + *cis*)%]_eq_ = 70 ± 1% [36].

**Figure 6:**
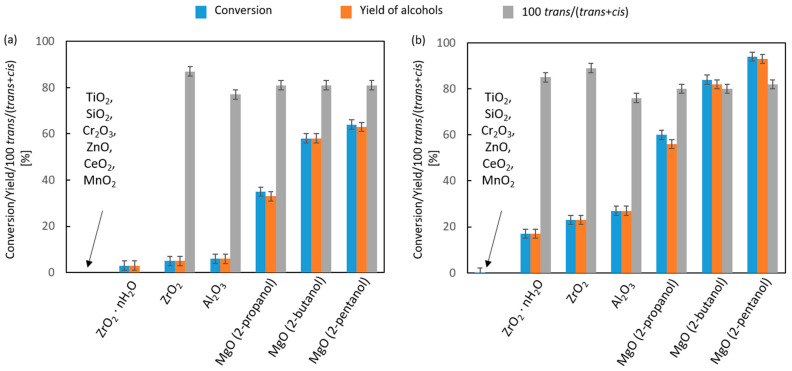
Liquid-phase transfer hydrogenation of 4-methylcyclohexanone with 2-propanol in the presence of metal oxide catalysts after (**a**) 1 h and (**b**) 6 h of reaction; D/A = 6. T_R_ = 355 K.

The results of the liquid-phase CTH of 4-methylcyclohexanone with 2-propanol in the presence of all ten metal oxides used as catalysts in the previous part of this study are summarized in Figure 6. It was found that 4-methylcyclohexanone is a less reactive hydrogen acceptor than 4-*t*-butylcyclohexanone because the determined conversion values of the first ketone were clearly lower than those achieved in the case of the second one. Six of the ten metal oxides used as catalysts were inactive in the CTH reaction, namely, SiO_2_, Cr_2_O_3_, ZnO, TiO_2_, MnO_2_, and CeO_2_. In their case, after 6 h of reaction, the conversion achieved was lower than 0.2%. Among the four most active catalysts, three showed moderate activity measured by conversion between 10 and 30% after 6 h of reaction. These were as follows (the activity is given in brackets): ZrO_2_· nH_2_O (17%) < Al_2_O_3_ (23%) < ZrO_2_ (27%). In their presence, 4-methylcyclohexanols were formed with a selectivity of 100%. The highest conversion (60%) was achieved after 6 h for the MgO catalyst. However, the selectivity to 4-methylcyclohexanols was reduced to 93% due to the aldol condensation of 4-methylcyclohexanone with acetone. MgO was further tested with this acceptor and the results are given in Table 7.

For the group of the most active catalysts, the diastereoselectivity value of the reaction to the *trans* isomer was in each case higher than the reference value (Table 7). Changing the hydrogen donor from 2-propanol to 2-butanol and 2-pentanol resulted in an increase in the reaction temperature from 355 to 373 and 392 K, respectively. Under these conditions, there was a significant increase in the conversion of 4-methylcyclohexanone after 6 h of reaction from 60 to 84 and 94%, respectively, with a very slight increase in selectivity to the mixture of 4-methylcyclohexanols and unchanged diastereoselectivity to the more stable diastereoisomer. The transfer hydrogenation reaction in the vapor phase was performed for two 2-propanol to 4-methylcyclohexanone molar ratios, namely, 3 and 6, in the temperature range 473–623 K (Table 7). In the case of both molar ratios, high ketone conversions of 84–85 and 95–97% and selectivity to 4-methylcyclohexanols of 97 and 95% were observed for D/A values of 3 and 6, respectively.

In order to check the influence of time on the activity, selectivity, and diastereoselectivity of MgO in the transfer hydrogenation reaction from 2-propanol to 4-methylcyclohexanone, a time-on-stream test was performed at a temperature of 473 K (Supplementary Materials, Figure S2). It was shown that both the activity and selectivity of the formation of the mixture of 4-methylcyclohexanols in the reaction, as well as the diastereoselectivity of the formation of the *trans* diastereoisomer, remain unchanged during 6 h of the test. The value of the determined diastereoselectivity is slightly lower than the reference value [36], but this change is related to the higher reaction temperature in our studies compared to the temperature given in the literature. Studies on the transfer hydrogenation of the remaining regioisomeric x-methylcyclohexanones, where x = 2 or 3, were carried out only for the two previously most active catalysts, i.e., Al_2_O_3_ and MgO, and were limited to the liquid-phase mode (Table 8 and Figure S3). According to the literature data on the composition of the 3-methylcyclohexanol mixture after the reduction of 3-methylcyclohexanone, the reference value [100 *trans/(trans + cis)*%]_eq_ is 22 ± 1% [36], whereas, for 2-methylcyclohexanone, the fraction of *trans*-2-methylcyclohexanols exceeds 94% [36]. 

MgO showed higher activity than Al_2_O_3_ in the CTH of 3-methylcyclohexanone; after 6 h, 76 and 56% conversions were recorded for the former and the latter catalysts, respectively. In the case of Al_2_O_3_, the value of diastereoselectivity to *trans* 3-methylcyclohexanol is equal to the reference value (21–22%). However, the composition of the mixture of 3-methylcyclohexanols formed in the reaction involving MgO was richer in the more stable *cis* diastereomer. This effect decreased when the reaction temperature increased to 373 K due to the change in the hydrogen donor from 2-propanol to 2-butanol and disappeared after a further increase in the reaction temperature to 392 K (2-pentanol). The presence of a methyl substituent in position 2 in relation to the carbonyl group of the cyclohexanone molecule results in the appearance of a steric hindrance for the reduction in the latter group. Indeed, the CTH of 2-methylcyclohexanone led to lower ketone conversions, 6 and 44% after 6 h for Al_2_O_3_ and MgO, respectively. It is noteworthy that there is a very significant difference in the diastereoselectivity of the reaction in the presence of these oxides. With MgO, mainly *trans* 2-methylcyclohexanol is formed (>96%), and, in the presence of Al_2_O_3_, the formation of a nearly equimolar mixture of 2-methylcyclohexanols (51–61%) was observed. An increase in the reaction temperature to 373 K, as a result of changing the donor to 2-butanol, causes only a slight increase in ketone conversion to 50%, with a simultaneous decrease in diastereoselectivity described by the value of 85–89%.

#### 2.2.3. Liquid- and Vapor-Phase Transfer Hydrogenation of Bicyclic Ketones: 2-Norbornanone, Camphor, and Fenchone

**Scheme 3:**
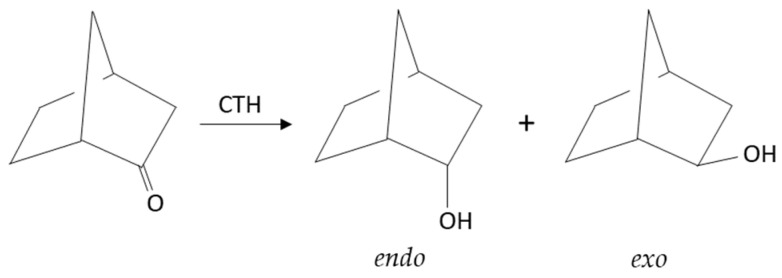
Transfer hydrogenation of 2-norbornanone.

**Scheme 4:**
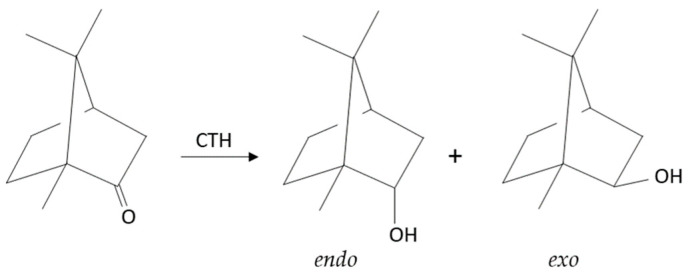
Camphor and its reduction products in CTH.

**Scheme 5:**
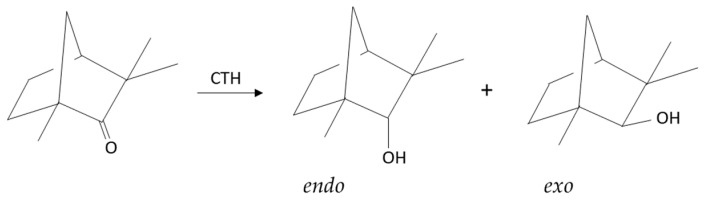
Transfer hydrogenation of fenchone.

The third part of our research is devoted to the Catalytic Transfer Hydrogenation of three bicyclic ketones: 2-norbornanone, camphor, and fenchone, in which the methylene bridge between the first and fourth carbon atoms of the 2-cyclohexanone ring stiffens the entire molecule. Moreover, in the case of the last two ketones, three methyl substituents located in different places in their molecules generate steric hindrances with different effects on both the reactivity of the ketones and the stereochemistry of the reduction of their carbonyl groups. The transfer hydrogenation of these three ketones is illustrated in Scheme 3, Scheme 4 and Scheme 5.

The thermodynamic equilibrium compositions of diastereomeric alcohols obtained as a result of the reduction of the three ketones are given in the literature [36,38,39]. In the case of 2-norbornanone, the thermodynamically more stable alcohol is *exo* 2-norbornanol; its fraction in the alcohol mixture is given by the expression [100 *exo*/(*exo + endo*)%]_eq_ = 79 ± 1% [36]. In contrast, as a result of the reduction of the remaining two ketones, the dominant alcohols are *endo* stereoisomers, and, hence, the following expressions are used for these two: [100 *endo*/(*exo + endo*)%]_eq_. The reference values for them are 70 ± 1% in the case of camphor [38] and 72% for fenchone [39], respectively. Unfavorable strains and angles in the alcohol molecule, resulting from the reduction of its parent ketone, during which the hybridization of the carbon–oxygen bond changes from sp^2^ to sp^3^, may be responsible for its lower stability compared to the second stereoisomer [23]. The literature describes successful attempts to explain the stereochemistry of the reduction of the carbonyl group of ketones depending on their structure based on two distinguished factors: steric approach control (SAC), i.e., the result of the competition between the attack of the hydride anion from the less crowded side of the carbonyl group and the attack of the hydride anion from the more eclipsed side of this group, and product development control (PDC), i.e., the thermodynamic stability of the formed alcohols [21]. The analysis of the alcohol compositions formed by the reduction in various monocyclic ketones with a carbonyl group without functional groups in its close proximity, such as 4-methylcyclohexanone, shows that the PDC factor dominates the reaction, and more stable thermodynamic equatorial alcohols are formed. On the other hand, the SAC factor largely determines the stereochemistry of the reduction in rigid, crowded bicyclic systems, such as camphor. As explained by Brown and Muzzio, the difference in the composition of the reduction products of the two groups of ketones results from the flexibility of the structures of the former [23]. Monocyclic ketones without bulky substituents have very flexible structures, capable of minimizing steric effects that appear during the reduction. Bicyclic ketones, on the other hand, are characterized by rigid, crowded structures that make access to the carbonyl group difficult. As a result of the reduction, two diastereomeric alcohols are formed; however, their mutual ratio is not determined by their thermodynamic stability but by the ease of access of the reducing agent to the carbonyl group from the less crowded side. However, it is not a rule that for flexible systems, only the PDC (thermodynamic) factor decides the course of the reaction, whereas, for bicyclic systems, it is only the SAC (steric) factor. The PDC factor is important for both systems. However, the SAC factor dominates in the reduction in rigid bicyclic systems more than in the reduction in flexible monocyclic systems. It is worth mentioning that the SAC factor also has a dominant effect during the reduction in monocyclic systems with a crowded carbonyl group [21]. As mentioned earlier (Section 2.2.3), *exo* 2-norbornanol is thermodynamically more stable compared to its *endo* isomer.

The results of our studies on the liquid-phase CTH of 2-norbornanone with 2-octanol in the presence of the ten metal oxides are summarized in Table 9. The catalysts in Table 9 are arranged in order of increasing activity expressed in conversion values. It is worth noting that 2-norbornanone gave lower conversion values in the reaction with 2-octanol than 4-*t*-butylcyclohexanone. SiO_2_, CeO_2_, TiO_2_, MnO_2_, and ZnO were found to be inactive in the reduction of 2-norbornanone; i.e., their activity was below 0.2%, similarly to the case of the CTH of 4-*t*-butylcyclohexanone (Section 2.2.1). Chromium oxide showed moderate activity; in its case, after 6 h, 16% conversion was observed.

Among the active catalysts in this reaction, the most active was ZrO_2_·nH_2_O. In its presence after 6 h of reaction, 82% conversion was noted with 96% selectivity to the mixture of 2-norbornanols. Most importantly, for all catalysts in this group, the diastereoselectivity value of the reaction towards the *endo* isomer was noted in the range of 70–80%, whereas the reference value is only 21 ± 1%. The observed difference in the diastereoselectivity values of the reaction is ample evidence for the dominant influence of the SAC factor on the stereochemistry of the 2-norbornanone reduction. For further studies involving 2-norbornanone, the ZrO_2_·nH_2_O catalyst was selected as the most active system in this series.

**Figure 7:**
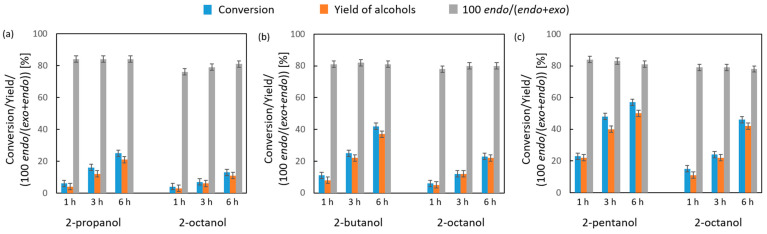
Results of the transfer hydrogenation of 2-norbornanone with secondary alcohols in the presence of ZrO_2_·nH_2_O as the catalyst at (**a**) 355 K, (**b**) 373 K, and (**c**) 392 K. D/A = 6.

In the next stage of the studies using 2-norbornanone as hydrogen acceptor, we determined the influence of the hydrogen donor structure on the CTH of one ketone with a set of secondary alcohols: 2-propanol, 2-butanol, 2-pentanol, and 2-octanol. In order to eliminate differences in the boiling points of individual donors and as their consequence differences in the observed reactivity, we performed activity measurements in the liquid phase at three temperatures: 355, 373, and 392 K, corresponding to the boiling points of 2-propanol, 2-butanol, and 2-pentanol, respectively (Figure 7). This method of conducting the reaction allowed us to determine the contribution resulting solely from the donor structure in the catalyst activity. Figure 7 shows the results of the comparison of the reactivity of three pairs of donors: 2-propanol and 2-octanol, 2-butanol and 2-octanol, and 2-pentanol and 2-octanol at the reaction temperatures determined by the boiling points of 2-propanol (355 K), 2-butanol (373 K), and 2-pentanol (392 K). It was shown that, for each pair of alcohols used as hydrogen donors in the reaction with 2-norbornanone, the alcohol with the lower number of carbon atoms is more reactive. Thus, at 355 K after 6 h of reaction, conversions of 25 and 13% were achieved, respectively, for the pair 2-propanol and 2-octanol, and a similar reactivity ratio, described by the ratio of 2 to 1, was noted at 373 K for the pair 2-butanol and 2-octanol. Only at the highest tested temperature (392 K) was the reactivity of 2-octanol after 6 h (46%) only somewhat lower than that of 2-pentanol (57%).

No influence of reaction temperature or hydrogen donor structure on the diastereoselectivity of the reaction was found (Figure 7). The value of this parameter fell within the range 78–84% in favor of the *endo* stereoisomer and was very far from the reference value (21 ± 1%). The invariance of the value of this parameter despite the use of hydrogen donors with different chain lengths indicates that, in the case of all donors used in the reaction, the reducing agent is a hydride anion.

**Figure 8:**
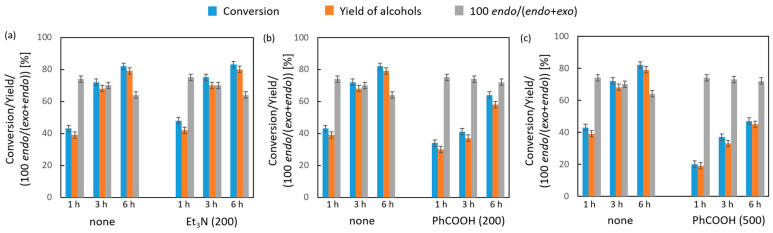
The influence of catalytic poisons: (**a**) 200 µmol·g^−1^ of triethylamine, and (**b**) 200 µmol·g^−1^ or (**c**) 500 µmol·g^−1^ of benzoic acid on the activity of ZrO_2_∙nH_2_O in transfer hydrogenation of 2-norbornanone with 2-octanol; T_R_ = 452 K, D/A = 6.

Continuing the investigation of the CTH of 2-norbornanone with 2-octanol in the presence of ZrO_2_·nH_2_O as the catalyst, we performed preliminary measurements of the activity of this catalyst treated with triethylamine and benzoic acid to selectively poison the surface Brønsted acidic (Et_3_N) and basic (PhCOOH) sites. The results of these measurements are shown in Figure 8. It was observed that poisoning of the Brønsted acid sites of the catalyst using Et_3_N at a concentration of 200 µmol·g^−1^ does not cause any changes in the activity, the selectivity towards alcohols, or in the diastereoselectivity of the reaction. In contrast, the application of benzoic acid onto the catalyst surface at a concentration of 200 µmol·g^−1^ results in a decrease in its activity in the reaction from a conversion value of 82% to 64% after 6 h. A further decrease in activity to 47% after 6 h was noted for the catalyst poisoned with benzoic acid at a concentration of 500 µmol·g^−1^. To sum up this part of this study, it should be stated that the Brønsted acid sites do not participate in the reaction, but the basic sites, i.e., oxide anions, do.

**Figure 9:**
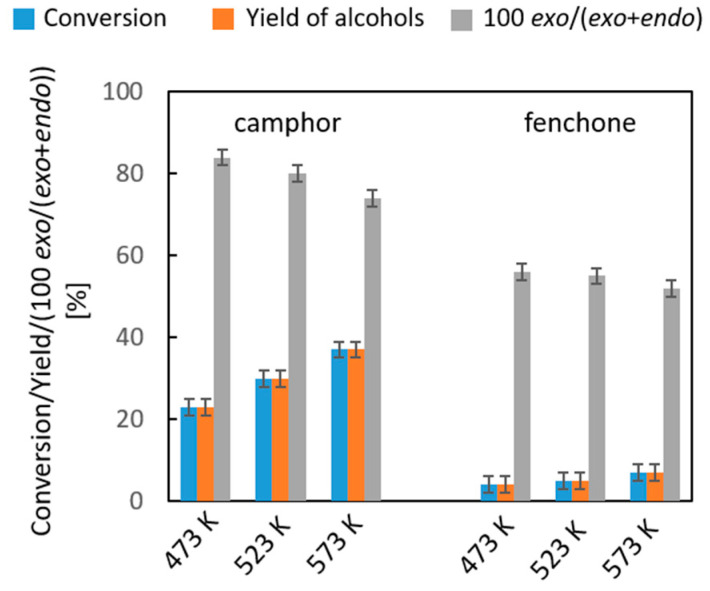
Vapor-phase transfer hydrogenation of camphor and fenchone with 2-propanol in the presence of MgO as a catalyst. D/A = 16.

The last part of this portion of this study was devoted to the liquid- and vapor-phase CTH of camphor and fenchone as hydrogen acceptors (Figure 9). No MgO activity in the liquid-phase CTH of camphor and fenchone was observed even after 6 h of reaction. Therefore, the reaction was transferred to the vapor phase, and catalytic tests were performed at temperatures of 473, 523, and 573 K at the donor–acceptor molar ratio of D/A = 16 (Figure 9). Under these conditions, the results of CTH were observed for both ketones. In both cases, the conversions increased with temperature: for camphor, from 23 to 37% and, for fenchone, from 4 to 7%. The low conversion of the latter is due to the presence of methyl substituents at both carbon atoms in positions α to the carbonyl carbon and, consequently, the occurrence of significant steric hindrance in the close vicinity of the carbonyl group. In the case of both ketones, 100% selectivity of reduction to alcohol mixtures was also found. The diastereoselectivity of reduction to the *exo* stereoisomers was higher than the reference values and decreased with temperature, from 84 to 74% for camphor and from 56 to 52% for fenchone.

#### 2.2.4. Liquid-Phase Transfer Hydrogenation of 2-Adamantanone

**Figure 10:**
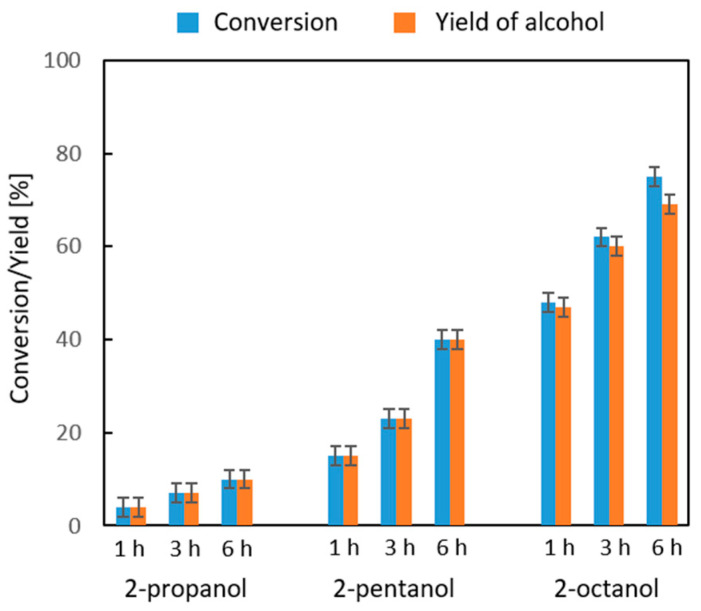
Liquid-phase transfer hydrogenation of 2-adamantanone with secondary alcohols in the presence of MgO as the catalyst; D/A = 16.

The last part of our studies is devoted to the CTH of a tricyclic ketone, namely, 2-adamantanone, with various secondary alcohols in the presence of MgO as the catalyst. Due to the high symmetry of the parent hydrocarbon molecule, the tested ketone does not offer the occurrence of two stereo-differentiated alcohols after the reduction of its carbonyl group to the carbinol group. In this experiment, we wanted to check how such a rigid molecule containing three condensed cyclohexane rings and with stress resulting only from the presence of a sp^2^ hybridized carbonyl carbon atom will undergo reduction in a transfer hydrogenation reaction. The results of this experiment are included in Figure 10. For each hydrogen donor used (2-propanol, 2-pentanol, and 2-octanol), the presence of 2-adamantanol in the post-reaction mixture was noted. Its fraction increased with temperature and reaction time. For the first two donors, the selectivity of this alcohol formation was 100% but only for 2-octanol; the presence of a cross-aldol condensation product between 2-adamantanone and 2-octanone was also noted. After 6 h of reaction, its yield was 6% with the yield of the main product equal to 69%.

## 3. Materials and Methods

### 3.1. Catalysts

Prior to activity experiments, grains of each of the studied catalysts were obtained either at the end of catalyst synthesis, in the case of the catalysts obtained from precursors (soluble salts) or by proper processing of powders of four commercial oxides (as per procedure details below).

Commercial SiO_2_ (Aerosil 250), Al_2_O_3_ (Alumina C), and TiO_2_ (P-25), all from Degussa (Frankfurt, Germany) in powder form, were mixed with redistilled water, and, after mixing, the resulting thick paste was left in closed containers at room temperature for 24 h. The obtained gels were transferred to porcelain cuvettes and dried under normal pressure in an oven at temperatures of 313, 353, and 393 K for 24 h at each temperature. The lumps were crushed, and a fraction with a grain size of 0.4–0.6 mm was separated, which was calcined at a temperature of 873 K in a stream of air for 1 h and for 5 h in a stream of nitrogen.

Cr_2_O_3_, CeO_2_, and ZrO_2_: As precursors of the oxides, the following compounds were used: Cr(NO_3_)_3_·9 H_2_O (purum p.a.), (NH_4_)_2_Ce(NO_3_)_6_ (puriss, p.a.), and ZrOCl_2_·6H_2_O (puriss. p.a.), all from Fluka AG, Buchs, Switzerland. A 15% aqueous solution of NH_3_ (25%, p.a., Polskie Odczynniki Chemiczne, POCh, Gliwice, Poland) was added dropwise to a solution of 200 g of each precursor in 2.5 dm^3^ of water over 3 h under constant stirring. After checking the completeness of precipitation, the precipitates were aged for 24 h at room temperature and then filtered under reduced pressure. The sediments were washed with water (20 times, 200 cm^3^). The obtained filter cakes were dried in air at temperatures of 333 and 393 K for 24 and 48 h, respectively. The lumps were crushed, and a fraction with a grain size of 0.4–0.6 mm was separated. The decomposition of hydroxides was carried out at a temperature of 873 K for 1 h in a stream of air and 5 h in a stream of nitrogen. The calcined samples of oxides were stored in tightly closed containers.

MgO: Magnesium oxide was obtained from commercial magnesium oxide (purum p.a., Reachim, Chişinåu, Moldova), which was treated with a nitric acid solution, and the obtained magnesium nitrate solution was purified as described in the study of [40]. In short, the magnesium hydroxide was obtained by adding a concentrated aqueous ammonia solution (25%, p.a., POCh, Gliwice, Poland) to the stirred solution of magnesium nitrate. The precipitate was washed by decantation with redistilled water (25 times, 1 dm^3^ portion of water each time) and dried at 313, 353, and 393 K for 24 h at each temperature. The soft lumps of the hydroxide were ground, and a powder was pressed in a hydraulic press using 10 MPa of pressure. The pellets were crushed, and a sieved fraction of 0.16–0.40 mm grains was calcined at 873 K as described for the abovementioned oxides.

ZnO: Powdered zinc oxide (purum p.a., Fluka AG, Buchs, Switzerland) was mixed with water to obtain a thick paste, which was left for 24 h at room temperature in a closed container. The paste was dried, crushed, and calcined in the same way as the previous preparations.

MnO_2_: 15 g of Mn(NO_3_)_2_·4 H_2_O (puriss. p.a., Fluka AG, Buchs, Switzerland) was placed in a quartz evaporating dish under a fume hood. The salt was heated with stirring with a thermometer to a temperature of 433–443 K, at which the evolution of NO_2_ ceased. After cooling, the solidified product was crushed and then washed with dilute (1:10) nitric acid. The precipitate was dried in an oven at 423 K for 6 h. The oxide prepared in this way was crushed to obtain a grain size of 0.1–0.2 mm, which was used in catalytic tests.

ZrO_2_·nH_2_O: A 4% aqueous solution of NaOH (puriss. p.a., POCH, Gliwice, Poland) was slowly added dropwise to a mechanically stirred solution of 200 g of ZrOCl_2_·6H_2_O (puriss. p.a., Fluka AG, Buchs, Switzerland) in 10 dm^3^ of water until pH = 6.8 was reached. The suspension was left for 48 h at room temperature, then filtered and washed until the chloride ions disappeared in the filtrate (20 times 200 cm^3^). The filter cake was dried at room temperature for 10 h and then at 353 K for 10 h. The product was crushed, separating a fraction of grains (0.4–0.6 mm), which was calcined at 573 K for 5 h in a stream of air and used for catalytic tests.

### 3.2. Hydrogen Acceptors

Commercial 2-methylcyclohexanone (99%, Aldrich, Poznań, Poland), 3-methylcyclohexanone (>97%, Fluka AG, Buchs, Switzerland), and 4-methylcyclohexanone (99%, ROTH, Karlsruhe, Germany) were purified by distillation under reduced pressure, and the middle fractions were taken. Their purities after distillation, as determined by GC analysis, were as follows: 98.4, 98.7, and 99.4% for 2-methyl-, 3-methyl-, and 4-methylcyclohexanone, respectively.

4-*t*-Butylcyclohexanone (99%, Aldrich, Poznań, Poland) was distilled twice under reduced pressure, and a colorless (snow-white) solid ketone was obtained, with purity of 99.6% (GC), and m.p. of 323 K.

*trans* 4-*t*-Butylcyclohexanol (99.1% GC), m.p. 420–1 K (exp.), 419–420 K (lit. [35]), and *cis* 4-*t*-butylcyclohexanol (98.0% GC), m.p. 354–5 K (exp.), 355–356.5 K (lit. [23]) used as GC standards were obtained by reduction of 4-*t*-butylcyclohexanone, according to procedures described elsewhere [35,41].

2-Norbornanone (bicyclo[2.2.1]heptan-2-one) was synthesized according to the procedure described in the literature [42]. In the first step, *exo* 2-norbornyl formate was obtained by reacting norbornene (98%, Aldrich, Saint Louis, MO, USA) with formic acid (98%, BDH, London, UK). In the second step, the obtained ester, dissolved in acetone, was oxidized with an aqueous solution of CrO_3_ in sulfuric acid. The crude product was separated, washed with a K_2_CO_3_ solution, dried, and distilled under normal pressure. The fraction boiling in the range of 443–444 K was collected. The product solidifies in the form of a colorless crystalline mass, m.p. of 369–370 K (exp), 370–371 K (lit. [42]), purity of 98.9% (GC), and yield of ketone 78%.

*exo* 2-Norbornanol was obtained by hydrolysis of *exo* 2-norbornyl formate with a water-ethanolic KOH solution [42]. For this purpose, the reaction mixture was heated under reflux for 4 h and cooled, and the product was extracted with diethyl ether. After drying the ethereal extract over anhydrous K_2_CO_3_, the solvent was evaporated, and the residue was distilled under normal pressure. The fraction with the boiling point between 450 and 452 K was collected (it solidifies easily upon cooling to room temperature, m.p. of 400–401 K), with a purity of 98.4% (GC) and yield of 76%.

Camphor (1,7,7-trimethyl bicyclo[2.2.1]heptan-2-one) (natural, BDH Chemicals Ltd., Poole, UK) was sublimed twice under normal pressure at around 443 K. Purity of the product was 99.4% (GC).

Fenchone (1,3,3-trimethyl bicyclo[2,2,1]heptan-2-one), ((1R)-(-) fenchone, Aldrich, Poznań, Poland) was distilled under reduced pressure, and a middle fraction boiling at 361 K/33 hPa was taken. Purity was 98.5% (GC).

2-Adamantanone (tricyclo[3.3.1.1^3,7^]decan-2-one) (99%, Aldrich, Poznań, Poland) was used as received.

### 3.3. Hydrogen Donors

Four aliphatic secondary alcohols: 2-propanol (puriss, POCh, Gliwice, Poland), 2-butanol (99%), 2-pentanol (98%), and 2-octanol (97%), all from Aldrich (Poznań, Poland), were purchased. Their purification was described in our previous work [20]. The final purities (GC) of the distillates were as follows: 2-propanol (99.8%), 2-butanol (99.4%), 2-pentanol (99.0%), and 2-octanol (98.4%). The alcohols were stored over freshly dehydrated 4A type molecular sieves in tightly closed containers.

### 3.4. Liquid-Phase Catalytic Activity Measurements

A weighed sample of an oxide (250 ± 5 mg) was introduced into a one-piece cylindrical glass reactor, equipped with a condenser, followed by a magnetic bar, the hydrogen donor, hydrogen acceptor, usually 5 millimoles, and *t*-butylbenzene as an internal standard. The reactor was heated in a silicon oil bath to a temperature 20 degrees higher than the boiling point of the donor to ensure ebullition of the solution. Samples of post-reaction mixtures were first centrifuged in order to separate the catalyst and then analyzed by GC. In experiments with poisoned catalysts, first, a magnetic bar was placed in the reactor; then, the catalyst was poured in, a hydrogen donor was added, and the poison was dosed. The content of the reactor was stirred for 5 min, and, then, the hydrogen acceptor and internal standard were added.

### 3.5. Vapor-Phase Catalytic Activity Measurements

A weighed sample of an oxide (250 ± 5 mg) was placed in a fixed-bed tubular glass reactor in a stream of nitrogen (50 cm^3^ min^−1^). After heating the reactor in the electric furnace to the set temperature, the reactant mixture containing *t*-butylbenzene was dosed using a microdosing pump with the Liquid Hourly Space Velocity (LHSV) of 3 cm^3^ per 1 g of catalyst per hour. The reaction products were collected in a receiver cooled to 0 °C in an ice-water bath. The post-reaction mixture obtained during the first 60 min of the reaction was discarded. Samples were taken within 30 min of the reaction, and their composition was determined by gas chromatography. In the double test, a fresh portion of the catalyst was used for activity measurements in the temperature range of 423–623 K, the spent catalyst was cooled in the stream of reagents to a temperature of 423 K, and the test was repeated.

### 3.6. Determination of the Composition of Post-Reaction Mixtures

The reaction products were analyzed using an HRGC KONIK (Barcelona, Spain) gas chromatograph equipped with a TRACER WAX capillary column (length 30 m, 0.25 mm i.d., Thermo Fisher Scientific, Dreieich, Germany) and a flame ionization detector. The compounds were identified by GC-MS (HP-6890N with a 5973N mass detector (Agilent, Santa Clara, CA, USA).

### 3.7. XRD Analysis

Powder X-ray diffraction patterns were recorded at room temperature on a Bruker D8 Advance diffractometer (Bruker AXS, Karlsruhe, Germany) equipped with a LYNXEYE position-sensitive detector (Bruker AXS, Karlsruhe, Germany) using Cu-Kα radiation (λ = 0.15418 nm). The data were collected in Bragg–Brentano (θ/θ) horizontal geometry (flat reflection mode) between 8° and 90° (2θ) in a continuous scan, using 0.03° steps with 2 s/step (total time 384 s/step). Data were collected under standard laboratory conditions.

### 3.8. Scanning Electron Microscopy Coupled with Energy-Dispersive X-Ray Spectroscopy (SEM-EDX)

The microscopes used for the imaging and composition determination was the PFIB Helios 5 DualBeam instrument (Thermo Fisher Scientific, Dreieich, Germany) and Prisma E (Thermo Fisher Scientific, Dreieich, Germany), equipped with SEM-EDX ChemiSEM^TM^. Technology. Prior to the measurements, the samples were taped onto a stub using carbon tape. Images of the surface were obtained in the electron beam mode with an Everhart–Thornley secondary electron detector with a voltage of 5 kV, a beam current of 13 pA, and a working distance of 3.7 mm at different magnifications. A magnification of 10,000 times was used to acquire the elemental maps for the O K, Al K, Cr K, Mn K, Zn K, Si K, Ce L, and Zr L lines using Prisma E with parameters suitable to obtain the following appropriate intensity counts: beam voltage = 15 kV, working distance = 10 mm, spot size = 5, dwell time = 200 µs.

### 3.9. Nitrogen Physisorption

The specific surface area of the catalysts was determined by nitrogen physisorption using the ASAP2020 device (Micromeritics Instrument Corporation, Norcross, GA, USA). Before the measurement, the samples were outgassed for 2 h at 423 K, and nitrogen adsorption was carried out at 77 K. The total specific surface area was determined using the S_BET_ (Brunauer–Emmett–Teller) adsorption isotherm model in the relative pressure range (p/p_0_) of 0.05–0.3.

### 3.10. DTA-TGA Measurements

The Differential Thermal Analysis (DTA)–Thermogravimetric Analysis (TGA) measurements of all studied catalysts were performed using approx. 125 mg of a sample. After an isothermal segment (30 min at 303 K), they were heated with a ramp of 10 K·min^−1^ to 1123 K in a flow (90 mL·min^−1^) of synthetic air (Multax, Stare Babice, Poland).

### 3.11. Strength and Concentration of Acidic/Basic Sites of Studied Metal Oxides

The strength of acidic and basic sites of studied oxides was determined using Hammett indicators. The following set of indicators was used (in parentheses the pK_BH+_ or pK_a_ values are given): chalcone (−5.6), dibenzylideneacetone (−3.0), crystal violet (0.8), methyl red (4.8), bromothymol blue (7.2), phenolphthalein (9.3), 2,4-dinitroaniline (15.0), 4-nitroaniline (18.4), diphenylamine (22.3), 4-chloroaniline (26.5) and triphenylmethane (33.0). To a sample of a catalyst (30 mg) placed in a vial, 0.5 cm^3^ of anhydrous toluene was added, followed by 0.15 cm^3^ of 0.1% solution of the appropriate indicator in anhydrous toluene, and the vial was sealed. After 24 h, the color of the grains of the catalyst was assessed and noted.

The concentration of acidic and basic sites on the surface of the catalysts was determined by treating the samples of metal oxides (200–250 mg), placed in Erlenmeyer flasks (100 cm^3^), with 20 cm^3^ of 0.010 M solutions of benzoic or n-butylamine in anhydrous toluene. After 24 h, 5 cm^3^ of a clear solution was taken, diluted with 10 cm^3^ of water, and the resulting mixture was titrated, in the presence of phenolphthalein as the indicator, with 0.010 M solutions of NaOH or HCl, respectively. The detailed procedures for the determination of the strength and the concentration of acidic and basic sites were described elsewhere [43].

## 4. Conclusions

The activity of the studied oxides correlated best with the acid/base properties of their surfaces. The comparison of the activity of the two catalysts with the highest surface areas, namely, ZrO_2_·nH_2_O and SiO_2_, showed that the high activity of the former is not caused by a high surface area but most likely by a much wider range of basic sites found on its surface. Moreover, the comparison of amorphous ZrO_2_·nH_2_O and crystalline ZrO_2_ revealed a higher activity of the former, which was not attributed to its lack of crystallinity (SiO_2_ was also amorphous and was inactive) but to the wider range of basic sites as revealed by Hammett indicator test results. Among the 10 selected metal oxides (Al_2_O_3_, CeO_2_, Cr_2_O_3_, MgO, MnO_2_, SiO_2_, TiO_2_, ZnO, ZrO_2_, and ZrO_2_·nH_2_O) only four of them (MgO, Al_2_O_3_, ZrO_2_, and ZrO_2_·nH_2_O) showed at least good or high activity in the liquid-phase CTH of 4-*t*-butylcyclohexanone with 2-octanol. The diastereoselectivity was dependent on the type of metal oxide; for ZnO and MnO_2_, it was lower than the reference value (78% *trans*), and, after 6 h of reaction, it was 58 and 61%, respectively. For the remaining oxides, this value was higher than 71%, and, most often, it was 80–90%. Lowering the reaction temperature due to changing the donor to 2-propanol (355 K) slightly decreased the conversion but increased the diastereoselectivity to 95–97%, except for Al_2_O_3_ (90%). In the case of CTH in the vapor phase, an increase in conversion (473 K, 95%) was observed with a significant decrease in diastereoselectivity to the *trans* isomer to 61–63%. Such a dramatic decrease in the reaction diastereoselectivity may be caused by a change in the reaction mechanism to a statistical attack of the ketone from the vapor phase onto the hydrogen donor adsorbed on the catalyst surface or by an increasing rate of the reverse reaction, which results in the equilibration of the composition of the 4-*t*-butylcyclohexanol mixtures.

Below, the remaining research results are presented for individual ketones divided into two groups: cyclic, bicyclic, and tricyclic ketones, along with the reference and experimental values of post-reduction product mixture compositions.

Cyclic ketones:

4-methylcyclohexanone—reference value 70 ± 1% (*trans*); liquid phase, MgO, Al_2_O_3_, ZrO_2_, and ZrO_2_·nH_2_O; [100 *trans*/(*trans* + *cis*)%] = 76–89%, vapor phase, MgO, conversion 97% (473 K), and [100 *trans*/(*trans* + *cis*)%] = 63%;

3-methylcyclohexanone—reference value 22 ± 1% (*cis*); liquid phase, [100 *trans*/(*trans* + *cis*)%] = 21% for Al_2_O_3_, and 7% for MgO;

2-methylcyclohexanone—reference value >94% (*trans*); liquid phase, conversion 6%, [100 *trans*/(*trans* + *cis*)%] = 51% for Al_2_O_3_, and [100 *trans*/(*trans* + *cis*)%] > 96% for MgO at 44% conversion.

Bicyclic and tricyclic ketones:

2-norbornanone—reference value 79 ± 1% (*exo*); liquid phase, [100 *exo*/(*exo + endo*)%] = 21% for Al_2_O_3_ at 64% conversion, 25% for MgO at 72% conversion, 27% for ZrO_2_ at 74% conversion, and 30% for ZrO_2_·nH_2_O at 82% conversion.

Camphor—reference value 30 ± 1% (*exo*); vapor phase, [100 *exo*/(*exo + endo*)%] = 74% for MgO at 37% conversion (573 K);

Fenchone—reference value 28% (*exo*); vapor phase, [100 *exo*/(*exo + endo*)%] = 52% for MgO at 7% conversion (573 K).

2-adamantanone—liquid phase, conversion 10% (2-propanol, 355 K), conversion 40% (2-pentanol, 392 K), and conversion 75% (2-octanol, 452 K).

The first, main conclusion resulting from the conducted research is that, in the presence of metal oxides as catalysts active in the CTH reaction of cyclic ketones, more thermodynamically stable diastereomeric alcohols are formed, the fraction of which exceeds the reference value. The second conclusion is that a significant majority of diastereomeric alcohols are formed, which are thermodynamically less stable as a result of CTH, from ketones having a rigid structure due to the presence of a methylene bridge connecting two carbon atoms of the cyclohexane ring and containing steric hindrance. The results obtained and conclusions drawn in this work confirm the rules known from the literature concerning the stereochemistry of the reduction of carbonyl compounds to alcohols in homogeneous systems. An issue that still requires explanation is the fact that there is a significant difference in the diastereoselectivity of reactions conducted with the same ketone, hydrogen donor, and catalyst, at similar temperatures, in the case of reactions conducted in the liquid and vapor phases. In light of the obtained results of the activity studies of the metal oxides used in the work in the CTH reaction and in connection with the acid–base properties of the surfaces of these oxides, the following conclusions can be drawn—the key to the high catalytic activity of the oxide is the high concentration of basic sites in connection with the low concentration of acid sites (MgO, ZrO_2_·nH_2_O, ZrO_2_, and Al_2_O_3_). SiO_2_ is inactive in the CTH reaction because the concentration of basic sites on its surface is close to zero. In the case of CeO_2_, high concentrations of both types of sites on its surface do not imply the catalytic activity of the oxide, most likely due to the low strength of basic sites.

## Figures and Tables

**Table 1:** Conditions of hydrogen transfer studies with cyclic and bicyclic ketones.

Cyclic Ketone	Reducing Agent	Catalyst	Mode of Reaction ^1^	Ref.
x-methylcyclohexanones	LiAlH_4_, NaBH_4_, Al(O*i*-C_3_H_7_)_3_, NaBH(OCH_3_)_3_	none	LP	[21]
	LiAlH(O-t-Bu)_3_	none	LP	[22]
	M^0^ + NH_3_ (liq) M^0^ = Li, Na, K, and Rb	none	LP	[28]
	LiAlH(OCH_3_)_3_	none	LP	[25]
	KHBPh_3_	none	LP	[26]
	2-propanol, 2-butanol	Al-free Sn-Beta zeolite	LP	[33]
menthone	LiAlH_4_, NaBH_4_, Al(O*i*-C_3_H_7_)_3_, NaBH(OCH_3_)_3_	none	LP	[21]
	Aliphatic alcohols	Al_2_O_3_ + 2 wt% Na	VP	[29]
	2-Propanol	ZrO_2_·nH_2_O	VP	[30]
4-*t*-butylcyclohexanone	LiAlH(O-t-Bu)_3_	none	LP	[22]
	KHBPh_3_	none	LP	[26]
	2-propanol	Gd(O*i*-C_3_H_7_)_3_	LP	[27]
	2-propanol	ZrO_2_·nH_2_O	VP	[30]
	2-propanol	Zeolite BEA (Si/Al = 12)	VP	[31]
	2-propanol, 2-butanol	Al-free Sn-Beta zeolite	LP	[32]
	Aliphatic alcohols	MgO	LP, VP	[19]
2-norbornanone	LiAlH(OCH_3_)_3_	none	LP	[25]
	KHBPh_3_	none	LP	[26]
	M^0^ + NH_3_ (liq) M^0^ = Li, Na, K, and Rb	none	LP	[28]
camphor	LiAlH(OCH_3_)_3_	none	LP	[25]
	KHBPh_3_	none	LP	[26]
	M^0^ + NH_3_ (liq) M^0^ = Li, Na, K, and Rb	none	LP	[28]
	2-Propanol	ZrO_2_·nH_2_O	VP	[30]

^1^—LP = liquid phase; VP = vapor phase.

**Table 2:** Specific surface area (S_BET_) and crystallite size of metal oxides used as catalysts.

Catalyst	2θ [°]/(hkl)	Crystallite Size [nm]	S_BET_ [m^2^·g^−1^]
Al_2_O_3_	67.1/(042)	12	103
MgO	42.9/(200)	12	100
ZrO_2_·nH_2_O	--	-- ^1^	235
ZrO_2_	28.2/(111)	18	32
Cr_2_O_3_	54.9/(116)	20	21
MnO_2_	37.3/(101)	22	2
SiO_2_	--	-- ^1^	253
TiO_2_	25.3/(101)	23 ^2^	35
	27.4/(110)	31 ^3^	
ZnO	36.2/(101)	33	4
CeO_2_	47.6/(220)	13	7

^1^—amorphous phase; ^2^—anatase; ^3^—rutile.

**Table 3:** Strength of acidic and basic sites of studied metal oxide catalysts.

Catalyst ^1^	H_0_				H_−_						
	−5.6	−3.0	0.8	4.8	7.2	9.3	15.0	18.4	22.3	26.5	33.0
Al_2_O_3_	−	**+**	**+**	**+**	**+**	−	−	−	−	−	−
MgO	−	−	−	−	**+**	**+**	**+**	**+**	**+**	**+**	−
ZrO_2_·nH_2_O	−	**+**	**+**	**+**	**+**	**+**	**+**	**+**	**+**	−	−
ZrO_2_	−	−	**+**	**+**	**+**	**+**	**+**	−	−	−	−
CeO_2_	−	−	**+**	**+**	**+**	**+**	**+**	−	−	−	−
TiO_2_	−	−	**+**	**+**	**+**	−	−	−	−	−	−
ZnO	−	−	−	**+**	**+**	**+**	−	−	−	−	−
SiO_2_	−	−	−	**+**	**+**	−	−	−	−	−	−

^1^—Cr_2_O_3_ and MnO_2_ were not tested using this method due to their intensive, dark colors.

**Table 4:** Concentration of acidic and basic sites on the surface of the studied catalysts.

Catalyst	Acidic Sites		Basic Sites	
	[µmol·g^−1^]	[µmol·m^−2^]	[µmol·g^−1^]	[µmol·m^−2^]
Al_2_O_3_	242.0	2.3	541.4	5.3
MgO	76.6	0.8	1174.8	11.7
ZrO_2_·nH_2_O	398.7	1.7	732.2	3.1
ZrO_2_	100.9	3.2	119.4	3.7
Cr_2_O_3_	179.7	8.6	51.0	2.4
MnO_2_	42.8	21.4	0.4	0.2
SiO_2_	321.1	1.3	3.4	~0
TiO_2_	147.8	4.2	114.5	3.3
ZnO	40.6	10.2	32.3	8.1
CeO_2_	121.4	17.3	186.6	26.7

**Table 5:** Liquid-phase transfer hydrogenation of 4-*t*-butylcyclohexanone with 2-octanol in the presence of metal oxide catalysts. Donor/acceptor molar ratio D/A = 6. T_R_ = 452 K.

Catalyst	Time [h]	Conversion [%]	Yield of Alcohols [%]	[100 *trans*/(*trans* + *cis*)] ^1^ [%]
SiO_2_	3	1	1	n.d.
	6	2 ^2^	2	n.d.
CeO_2_	3	2	2	n.d.
	6	4	4	n.d.
ZnO	3	5	5	58
	6	9	8	58
MnO_2_	3	5	5	60
	6	10	9	61
TiO_2_	3	6	6	86
	6	12	11	87
Cr_2_O_3_	3	12	12	83
	6	23	22	82
ZrO_2_	1	78	77	90
	3	88	87	89
ZrO_2_·nH_2_O	1	73	71	87
	3	92	89	86
Al_2_O_3_	1	91	74	87
	3	96	66	88
MgO	1	95	88	79
	3	98	86	71

^1^—[100 *trans/(trans + cis)%*]_eq_ = 78 ± 1% [36]; ^2^—in the absence of a catalyst, 0.2% conversion of 4-*t*-butycyclohexanone is noted after 6 h of reaction.

**Table 6:** Liquid-phase transfer hydrogenation of 4-*t*-butylcyclohexanone with 2-propanol in the presence of metal oxide catalysts. D/A = 6. T_R_ = 355 K.

Catalyst	Time [h]	Conversion [%]	Yield of Alcohols [%]	[100 *trans*/(*trans* + *cis*] ^1^ [%]
ZrO_2_	1	20	19	95
	3	47	45	96
ZrO_2_·nH_2_O	1	36	36	95
	3	70	69	95
Al_2_O_3_	1	47	47	91
	3	78	77	90
MgO	1	80 ^2^	79	97
	3	86	82	97

^1^—[100 *trans*/(*trans* + *cis*)%]_eq_ = 78 ± 1% [36]; ^2^—54% conversion after 15 min.

**Table 7:** Vapor-phase transfer hydrogenation of 4-methylcyclohexanone with 2-propanol in the presence of MgO catalyst. Influence of the donor–acceptor molar ratio on the course of the reaction.

D/A	T [K]	Conversion [%]	Yield of Alcohols [%]	[100 *trans*/(*trans + cis*)] ^1^ [%]
3	473	85	82	67
	523	84	81	65
	573	84	81	64
	623	84	81	63
6	473	97	92	63
	523	96	91	62
	573	96	91	60
	623	95	90	59

^1^—[100 *trans/*(*trans* + *cis*)%]_eq_ = 70 ± 1% [36].

**Table 8:** Liquid-phase transfer hydrogenation of 3-methylcyclohexanone with 2-propanol in the presence of Al_2_O_3_ and MgO as catalysts. D/A = 6. T_R_ = 355 K.

Catalyst	Time [h]	Conversion [%]	Yield of Alcohols [%]	[100 *trans*/(*trans + cis*)] ^1^ [%]
Al_2_O_3_	1	15	15	22
	6	56	55	21
MgO	1	68	64	5
	6	76	69	7
MgO ^2^	1	66	64	19
	6	86	82	18
MgO ^3^	1	80	78	22
	6	96	92	21

^1^—[100 *trans/(trans + cis*)%]_eq_ = 22 ± 1% [36]; ^2^—2-butanol as hydrogen donor, T_R_ = 373 K; ^3^—2-pentanol as hydrogen donor, T_R_ = 392 K.

**Table 9:** Liquid-phase transfer hydrogenation of 2-norbornanone with 2-octanol in the presence of selected metal oxide catalysts. T_R_ = 452 K, D/A = 6.

Catalyst	Time [h]	Conversion [%]	Yield of Alcohols [%]	[100 *endo*/(*endo + exo*)] ^1^ [%]
MO_x_^2^	6	<0.2	n.d.	--
Cr_2_O_3_	1	13	13	78
	6	16	16	77
Al_2_O_3_	1	18	14	80
	6	64	54	79
MgO	1	31	30	79
	6	72	67	75
ZrO_2_	1	32	30	79
	6	74	71	73
ZrO_2_·nH_2_O	1	43	39	74
	6	82	79	70

^1^—[100 *endo*/(*endo + exo*)%]_eq_ = 21 ± 1% [37]; ^2^ MO_x_ = SiO_2_, CeO_2_, TiO_2_, MnO_2_, or ZnO as the catalysts.

## Data Availability

The original contributions presented in this study are included in the article/Supplementary Materials. Further inquiries can be directed to the corresponding author.

## Supplementary Materials

The following supporting information can be downloaded at https://www.mdpi.com/article/10.3390/molecules30102153/s1, Scheme S1: Products of Catalytic Transfer Hydrogenation of the studied ketones; Figure S1: Thermal analysis results of (a) ZrO_2_, (b) ZrO_2_·n H_2_O, (c) SiO_2_, (d) MnO_2_, (e) MgO, (g) Cr_2_O_3_, (f) TiO_2_, (h) ZnO, (i) Al_2_O_3_, and (j) CeO_2_; Figure S2: Time-on-stream test for vapor-phase transfer hydrogenation of 4-methylcyclohexanone with 2-propanol in the presence of MgO catalyst. D/A = 6. TR = 473 K; Figure S3: Liquid-phase transfer hydrogenation of 2-methylcyclohexanone with 2-propanol in the presence of metal oxide catalysts (a) 1 h and (b) 6 h of reaction; D/A = 6. TR = 355 K.
