# Supplementary material for: Diastereoselective Transfer Hydrogenation of Cyclic and Bicyclic Ketones over Selected Metal Oxides as Catalysts
Source: Molecules. 2025 May 14;30(10):2153. doi: 10.3390/molecules30102153 (PMC12114447; doi:10.3390/molecules30102153)
Supplement: Supplementary file 1 [file molecules-30-02153-s001.zip › molecules-3586604-supplementary.pdf]

Supplementary Materials

Diastereoselective Transfer Hydrogenation of Cyclic and Bicyclic Ketones over Selected Metal Oxides as Catalysts

Marek Gliński *, Dorota Armusiewicz, Karolina Łukasik-Kwaśniewska, Michał Materowski, Adam Rułka, Ewa M. Iwanek (nee Wilczkowska) and Monika Kucharska

Warsaw University of Technology; marek.glinski@pw.edu.pl; pdkalisz@wp.pl; karo.lukasik@gmail.com; michal.materowski@gmail.com; rulka.adam@gmail.com; ewa.iwanek@pw.edu.pl; monika.wozniak25@vp.pl

*Correspondence: marek.glinski@pw.edu.pl; Tel.: (+48-22 234 7594)

Pages: 4
Schemes: 1
Figures: 3

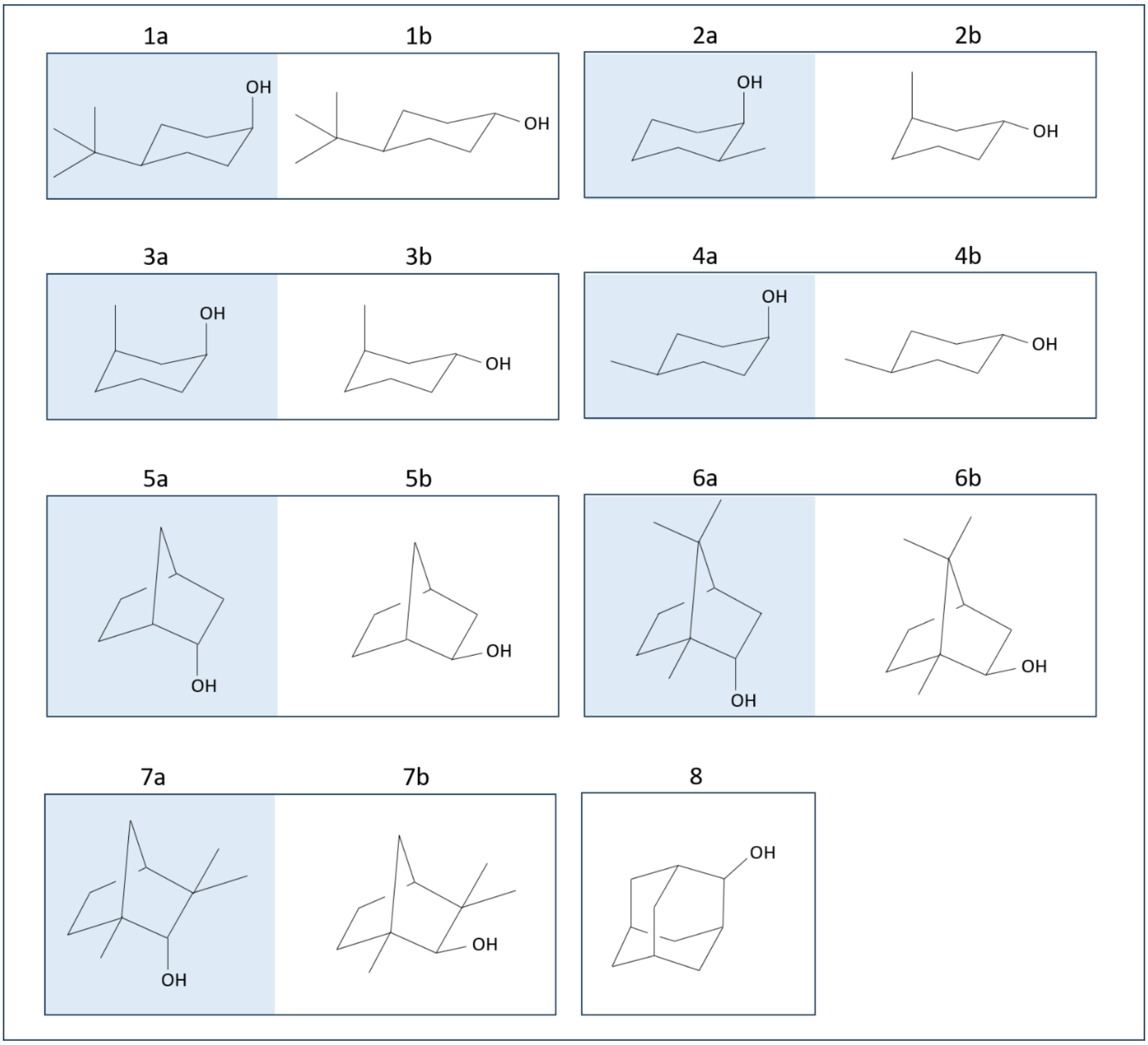

Scheme S1. Products of Catalytic Transfer Hydrogenation of the studied compounds: (1) 4-*t*-butylcyclohexanols (a) *cis*, (b) *trans*, (2) 2-methylcyclohexanols (a) *cis*, (b) *trans*, (3) 3-methylcyclohexanols (a) *cis*, (b) *trans,* (4) 4-methylcyclohexanols (a) *cis*, (b) *trans,*(5) 2-norbornanols (a) *endo*, (b) *exo*, (6a) borneol, (6b) isoborneol, (7) fenchols (a) *endo*, (b) *exo* and (8) 2-adamantanol.

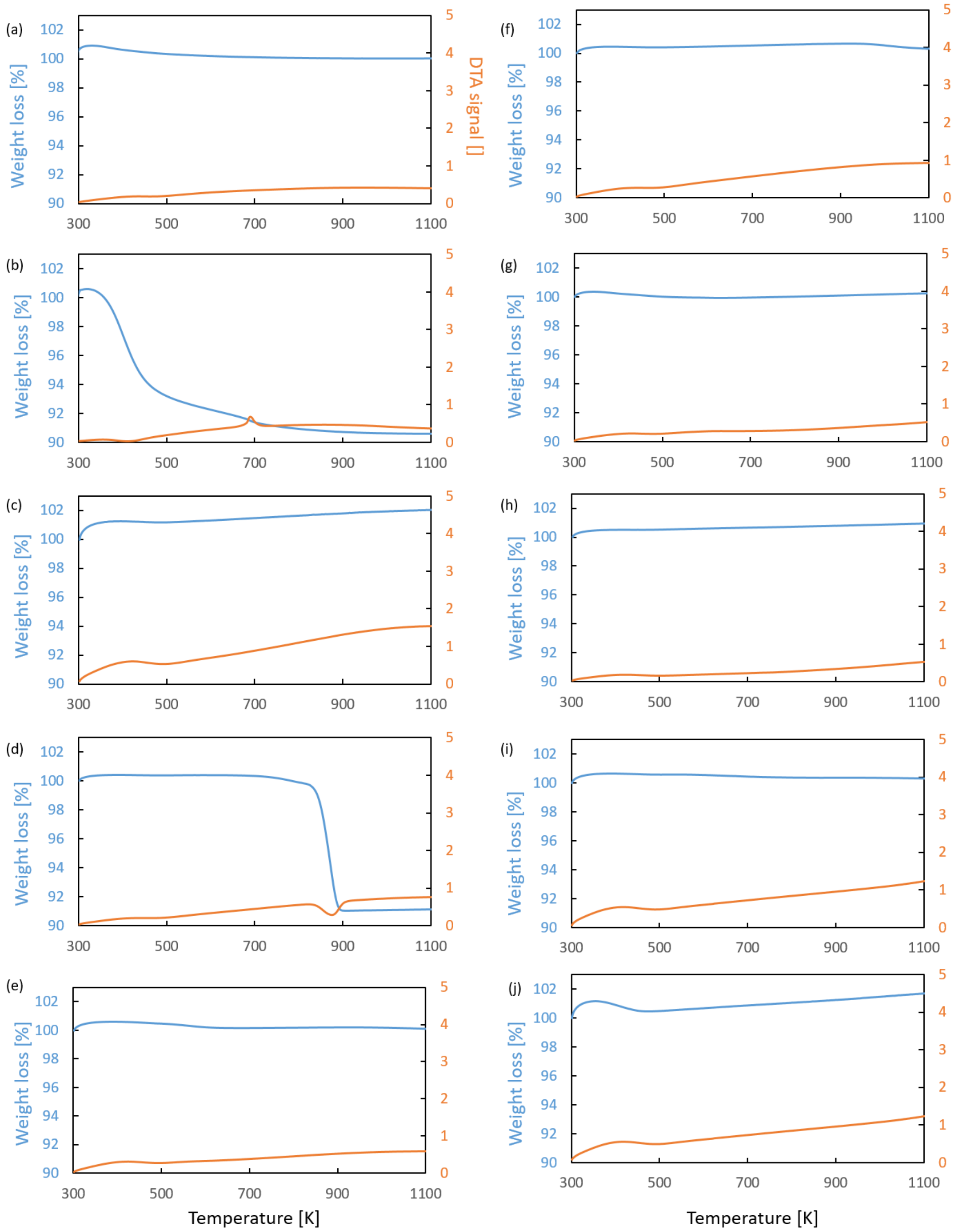

Figure S1. Thermal analysis results -weight loss (blue) and DTA curves (orange) of the studied catalysts: (**a**) ZrO_2, (**b**) $ZrO_2 \cdot n\ H_2O$, (**c**) SiO_2, (**d**) MnO_2, (**e**) MgO, (**f**) Cr_2O_3, (**g**) TiO_2, (**h**) ZnO, (**i**) Al_2O_3 and (**j**) CeO_2.

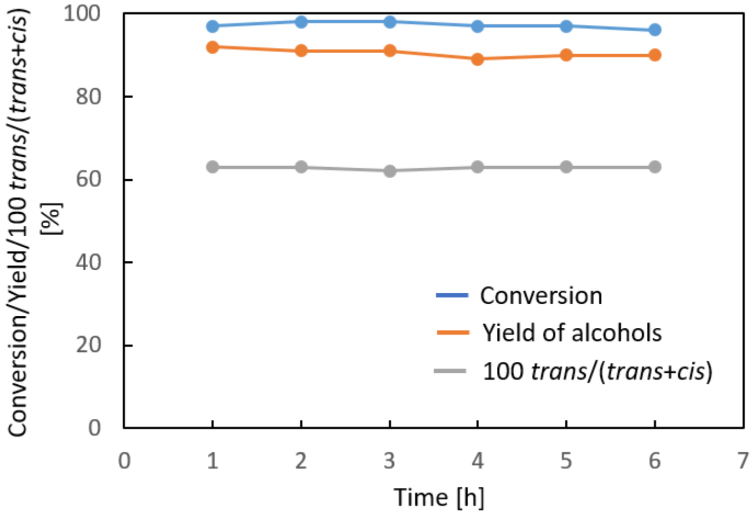

Figure S2. Time-on-stream test for vapor-phase transfer hydrogenation of 4-methylcyclohexanone with 2-propanol in the presence of MgO catalyst. D/A = 6. T_R = 473 K.

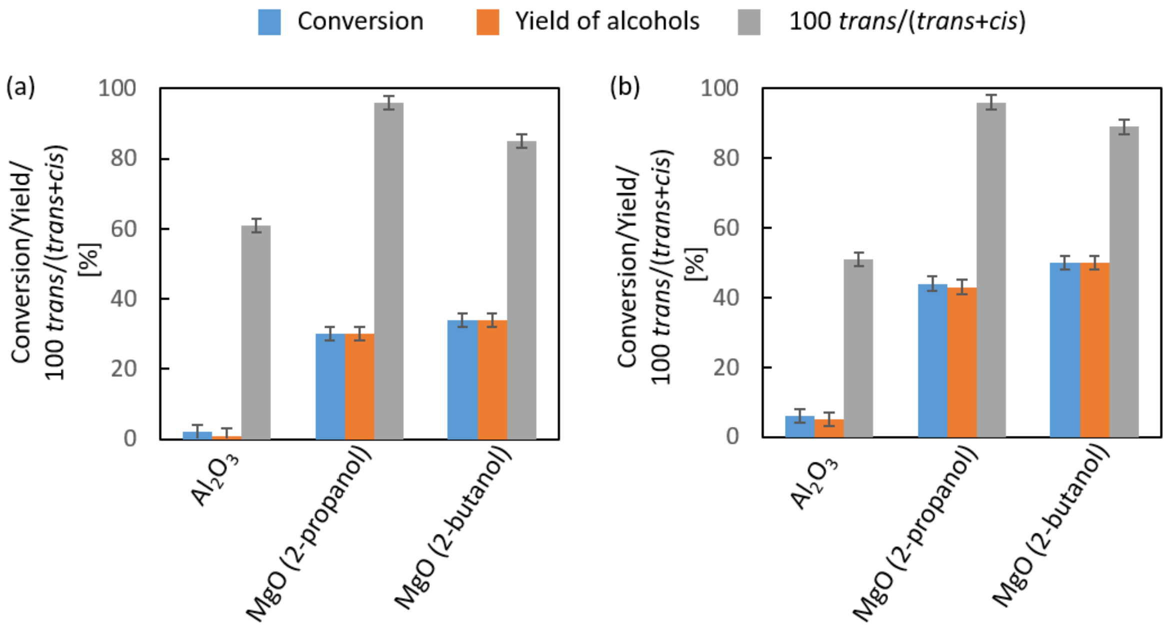

Figure S3. Liquid-phase transfer hydrogenation of 2-methylcyclohexanone with 2-propanol in the presence of metal oxide catalysts (**a**) 1 h and (**b**) 6 h of reaction; D/A = 6. T_R = 355 K.
